# Stigma/Style Cell-Cycle Inhibitor 1, a Regulator of Cell Proliferation, Interacts With a Specific 14-3-3 Protein and Is Degraded During Cell Division

**DOI:** 10.3389/fpls.2022.857745

**Published:** 2022-04-04

**Authors:** Edward J. Strini, Lígia T. Bertolino, Juca A. B. San Martin, Hebréia A. O. Souza, Francine Pessotti, Vitor F. Pinoti, Pedro B. Ferreira, Henrique C. De Paoli, Greice Lubini, Luiz-Eduardo Del-Bem, Andréa C. Quiapim, Mateus Mondin, Ana Paula U. Araujo, Nubia B. Eloy, Matteo Barberis, Maria Helena S. Goldman

**Affiliations:** ^1^Departamento de Biologia, Faculdade de Filosofia, Ciências e Letras de Ribeirão Preto, University of São Paulo, Ribeirão Preto, Brazil; ^2^PPG-Genética, Faculdade de Medicina de Ribeirão Preto, University of São Paulo, Ribeirão Preto, Brazil; ^3^Departamento de Botânica, Instituto de Ciências Biológicas, Universidade Federal de Minas Gerais, Belo Horizonte, Brazil; ^4^Departamento de Genética, Escola Superior de Agricultura Luiz de Queiroz, University of São Paulo, Piracicaba, Brazil; ^5^São Carlos Institute of Physics, University of São Paulo, São Carlos, Brazil; ^6^Departamento de Ciências Biológicas, Escola Superior de Agricultura Luiz de Queiroz, University of São Paulo, Piracicaba, Brazil; ^7^Systems Biology, School of Biosciences and Medicine, Faculty of Health and Medical Sciences, University of Surrey, Guildford, United Kingdom; ^8^Centre for Mathematical and Computational Biology, CMCB, University of Surrey, Guildford, United Kingdom; ^9^Synthetic Systems Biology and Nuclear Organization, Swammerdam Institute for Life Sciences, University of Amsterdam, Amsterdam, Netherlands

**Keywords:** cell division regulator, early mitosis, 14-3-3 interaction, nuclear shuttling, proteasome degradation, SCI1

## Abstract

The final shape and size of plant organs are determined by a network of genes that modulate cell proliferation and expansion. Among those, *SCI1 (Stigma/style Cell-cycle Inhibitor 1)* functions by inhibiting cell proliferation during pistil development. Alterations in *SCI1* expression levels can lead to remarkable stigma/style size changes. Recently, we demonstrated that *SCI1* starts to be expressed at the specification of the *Nicotiana tabacum* floral meristem and is expressed at all floral meristematic cells. To elucidate how SCI1 regulates cell proliferation, we screened a stigma/style cDNA library through the yeast two-hybrid (Y2H) system, using SCI1 as bait. Among the interaction partners, we identified the 14-3-3D protein of the Non-Epsilon group. The interaction between SCI1 and 14-3-3D was confirmed by pulldown and co-immunoprecipitation experiments. 14-3-3D forms homo- and heterodimers in the cytoplasm of plant cells and interacts with SCI1 in the nucleus, as demonstrated by Bimolecular Fluorescence Complementation (BiFC). Analyses of SCI1-GFP fluorescence through the cell-cycle progression revealed its presence in the nucleoli during interphase and prophase. At metaphase, SCI1-GFP fluorescence faded and was no longer detected at anaphase, reappearing at telophase. Upon treatment with the 26S proteasome inhibitor MG132, SCI1-GFP was stabilized during cell division. Site-directed mutagenesis of seven serines into alanines in the predicted 14-3-3 binding sites on the SCI1 sequence prevented its degradation during mitosis. Our results demonstrate that SCI1 degradation at the beginning of metaphase is dependent on the phosphorylation of serine residues and on the action of the 26S proteasome. We concluded that SCI1 stability/degradation is cell-cycle regulated, consistent with its role in fine-tuning cell proliferation.

## Introduction

Successful sexual plant reproduction depends on the proper development of the male and female reproductive organs, stamens, and pistil, respectively. The upper pistil, comprised of style and stigma, is responsible for receiving the pollen grains and providing the appropriate conditions for compatible pollen tube germination and directional growth (Goldman et al., 1994). Pistil organogenesis relies on the tight control of cell proliferation and differentiation, achieved through complex gene regulatory networks that are not fully understood. The identification and characterization of *SCI1* showed that this gene has a role in the network controlling cell proliferation in the upper pistil of *Nicotiana tabacum* (DePaoli et al., 2011) and *Arabidopsis thaliana* (DePaoli et al., 2014). Knockdown transgenic tobacco plants and Arabidopsis mutants display enlarged stigmas and elongated styles that contain more cells than plants with normal SCI1 transcript levels (DePaoli et al., 2011, 2014). Based on the evidence, we proposed that SCI1 is a tissue-specific regulator of cell proliferation (DePaoli et al., 2012). Recently, SCI1 was demonstrated to be specifically expressed in the proliferative cells of the *N. tabacum* floral meristem, which is terminated after style and stigma development (Cruz et al., 2021).

Stigma/style cell-cycle inhibitor 1 is a small protein (154 amino acids) containing 15 putative phosphorylation sites (NetPhos, ≥96%), suggesting that it may be regulated in a signal transduction pathway (DePaoli et al., 2011). To elucidate how SCI1 engages with the cell-cycle machinery, we have undertaken different approaches to identify SCI1 interaction partners. By analyzing the SCI1 interactome in tobacco stigmas/styles, we identified, among many other proteins, two 14-3-3 proteins of the Non-Epsilon group. 14-3-3s are conserved eukaryotic proteins that usually recognize proteins containing phosphoserine and/or phosphothreonine residues within specific sequence motifs (de Boer et al., 2012; Wilson et al., 2016). They are regulatory proteins with signaling roles in various cellular processes, such as regulation of hormonal signal transduction processes (Roberts, 2000; Aducci et al., 2002; Schoonheim et al., 2007; Chevalier et al., 2009; Denison et al., 2011; Keicher et al., 2017; Camoni et al., 2018; Lee et al., 2020), plant growth dependent on sugar signaling (Chen et al., 2019), and flowering transition (Mayfield et al., 2007; Paul et al., 2008; Jaspert et al., 2011; Collani et al., 2019). Their involvement in cell-cycle control (Laronga et al., 2000; van Hemert et al., 2001; Usui and Petrini, 2007; Grandin and Charbonneau, 2008; Gardino and Yaffe, 2011; Bokros et al., 2016; Gryaznova et al., 2016; Pennington et al., 2018) is well studied in yeast, animals and cancer research, but largely unknown in plants. In budding yeast, there are only two genes encoding 14-3-3s, BMH1 and BMH2. Mutants lacking both genes, which are involved in regulating cell division, are lethal. Interestingly, these mutants can be rescued by expressing the 14-3-3 counterparts from Arabidopsis, among which the isoforms At14-3-3 Phi and At14-3-3 Upsilon (van Heusden et al., 1996), both from the Non-Epsilon group (see below). This suggests that 14-3-3 proteins may be involved in regulating cell division also in plants.

Phylogenetically, plant 14-3-3 protein family can be divided into the Epsilon and the Non-Epsilon groups (Ferl et al., 2002), based on their amino acid sequences and gene structures. The Epsilon group is considered to contain the ancestral 14-3-3s undertaking essential functions, while the 14-3-3s from the Non-Epsilon group would assume more specialized roles (Jaspert et al., 2011; Hloušková et al., 2019). 14-3-3 proteins can form homo and heterodimers (Jones et al., 1995; Chaudhri et al., 2003), and it has been suggested that each type of dimer could undertake diverse functions (Paul et al., 2012). There is certain specificity in 14-3-3 dimerization and not all homodimers and possible heterodimers are formed (Pallucca et al., 2014; Lin et al., 2021). The 14-3-3 dimer can interact with two different sites of a single protein or simultaneously interact with two distinct proteins (Yaffe, 2002; Chevalier et al., 2009). At the genomic level, 13 genes encode 14-3-3 proteins in tomato (Cao et al., 2016) and Arabidopsis, in addition to two pseudogenes in the latter genome (DeLille et al., 2001; Rosenquist et al., 2001). In *N. tabacum*, 17 transcript sequences encoding 14-3-3 proteins were identified (Konagaya et al., 2004; Lozano-Durán and Robatzek, 2015), and two of these correspond to alternative transcripts of the same gene. Considering the number of genes encoding the monomeric subunits and the possibility of forming different combinations of homo- and heterodimers, 14-3-3 proteins can assume many functions and specificities of client proteins. The binding of 14-3-3 dimers can cause a conformational change of the client proteins, stimulating or inhibiting their activity or modifying their stability (Ferl et al., 2002). Additionally, 14-3-3 binding can facilitate the interaction between two proteins or change their subcellular localization (van Heusden, 2009; reviewed in Chevalier et al., 2009; Gökirmak et al., 2010). Thus, plant 14-3-3 proteins can be considered nodes in signaling networks and establish a crosstalk platform in diverse pathways (Ferl, 2004; Oecking and Jaspert, 2009).

Here, we investigated the cellular and molecular mechanisms through which SCI1 may engage in cell-cycle control and regulate cell proliferation. We report the identification and characterization of SCI1 interaction with 14-3-3D from *N. tabacum*. We show that when chromatin starts condensation (G2/prophase), 14-3-3D moves from the cytoplasm to the nucleus, where it interacts with SCI1. SCI1 is degraded by the proteasome at late prophase/prometaphase, possibly due to its interaction with 14-3-3D, since mutations at key serine residues of SCI1 stabilize the protein during metaphase. This behavior is consistent with a role for SCI1 as an inhibitor of cell proliferation, which must be degraded at mitosis to allow proper cell division.

## Materials and Methods

### Plant Material

*Nicotiana tabacum* L. cv. Petit Havana SR-1 and *Nicotiana benthamiana* seeds were sown on Bioplant® substrate with vermiculite, irrigated from below, and cultivated in an environmental growth chamber Weiss-Gallenkamp (55% humidity, 16-h day/8-h night regime, and 22°C). In addition, wild-type and transgenic *N. tabacum* plants were also grown in a greenhouse under standard conditions in Ribeirão Preto—SP, BRAZIL (Latitude—21° 10′24″ S, Longitude—47° 48′24″ W, with an average temperature of 22°C in winter and 27°C in summer; the difference in day length between summer and winter is less than 2 h) for collecting floral tissues (for protein and RNA extractions). Samples were immediately used or frozen in liquid nitrogen and stored at −70°C.

### Y2H cDNA Library Construction

Total RNA was extracted from stigmas/styles of *N. tabacum* flowers at stages 1–11 (Koltunow et al., 1990) with Trizol reagent (Invitrogen). mRNA was purified with GenElute mRNA Miniprep (Sigma), and cDNA synthesis was performed with CloneMiner II cDNA Library Construction (Invitrogen) following the manufacturer’s instructions. Briefly, biotin-attB2-Oligo(dT) primer was used for reverse transcription reaction followed by second-strand synthesis and attB1 adapter ligation to the 5′ end of double-stranded cDNAs. The resulting cDNAs were size-fractionated by column chromatography, cloned in pDONR™222 (Invitrogen) with BP Clonase II enzyme mix (Invitrogen), and transformed into DH10B cells. The quality and the average insert cDNA size were estimated based on electrophoresis analyses of DNA from 24 transformants. Then, BP reactions were performed until 5 × 10^6^ clones were obtained. The plasmid DNA of the pDONR™ 222 clones (clone library) was purified, subcloned in pDEST22 (Invitrogen) vector with LR Clonase II Enzyme Mix (Invitrogen), and transformed into DH10B cells. The average insert cDNA size and the library quality were estimated, and LR reactions were performed until 5 × 10^6^ clones were obtained. Finally, plasmid DNA from the pDEST22 clones was extracted as a pool, composing the final yeast two-hybrid (Y2H) cDNA library.

### Screening of Y2H cDNA Library and Binary Assays of Candidate Clones

The screening of the Y2H cDNA library was carried out using ProQuest™ Two-Hybrid System and Gateway Technology kit (Invitrogen). Yeast strain PJ69-4a (MATa; trp1-901 leu2-3,112 ura3-52 his3-200 gal4 (deleted) gal80 (deleted) LYS2::GAL1-HIS3 GAL2-ADE2 met2::GAL7-lacZ) cells, previously transformed with pDEST32-SCI1 plasmid and tested for self-activation, were transformed with 1 μg of DNA of the Y2H library. The transformation procedure was repeated 30 times to achieve approximately 1–2 × 10^6^ yeast transformants. The transformed yeast cells were plated on selective Synthetic Complete (SC) medium (lacking leucine, tryptophan, and histidine) and incubated for 2 days at 30°C followed by 1–2 days at room temperature. During that period, the appearing colonies were scored and labeled. The HIS3 positive yeast colonies were organized in 96 well microtiter plates, used for glycerol stock preparation and LacZ reporter gene assay. Plasmid DNA from each positive yeast colony was extracted and individually transformed into DH10B cells. Plasmid DNA from *Escherichia coli* transformants was analyzed by restriction digestion. Distinct AD plasmids from a single *E. coli* transformation were individually introduced in PJ69-4a cells bearing pDEST32-SCI1 or pDEST32-empty vector and tested for histidine auxotrophy (binary assay). Different concentrations of 3′AT (3-Amino-1,2,4-triazole) were added on a selective medium in the case of self-activation. All plates of Y2H binary assays were performed in 96-well format (for an example, see Supplementary Figure S1), which included a set of the negative and positive controls of the ProQuestTM Two-Hybrid System and Gateway Technology kit (Invitrogen). Finally, the AD plasmids presenting growth along with pDEST32-SCI1, but not with empty vector (self-activation control), were sequenced, analyzed by the BLAST program,^1^ and annotated as positive interactors.

### Cloning Procedures

The complete coding sequences (CDSs) of 14-3-3A1 (accession number AB119466) and 14-3-3D2AS (AB119473) were amplified by PCR from the Y2H cDNA library clones H6.1 and D9.4, respectively. *Nicotiana tabacum* Wee1 coding sequence was amplified by PCR from stigma/style cDNAs. All primers used for amplification and cloning are listed in Table 1. CDSs were cloned into the pDONR221 entry vector using BP Clonase (Invitrogen). pENTRY-SCI1 was produced in previous work (DePaoli et al., 2011). *Escherichia coli* DH10B electrocompetent cells were used for all cloning procedures. The CDSs were transferred to the appropriate destination vectors: pK7FWG2 for GFP fusion in plants (Karimi et al., 2002); pDEST32 and pDEST22 (Invitrogen) for Y2H; and pDEST15 and pDEST17 (Invitrogen) for *E. coli* expression with appropriate tags, using LR clonase (Invitrogen). The construction of the Bimolecular Fluorescence Complementation (BiFC) vectors, based on pK7m34GW and pH7m34GW and building blocks (Karimi et al., 2007), was done using LR Clonase II Plus (Invitrogen). Site-directed mutagenesis in SCI1 serines (shown in Supplementary Figure S2) was done in two steps, using the QuikChange™ Site-Directed Mutagenesis kit (Stratagene), according to manufacturer’s instructions, and the primers listed in Table 1. All constructions were sequenced to verify undesired PCR or cloning errors and confirmed the correct reading frame in the translational fusions.

**Table 1:** Primers used to amplify the coding regions of *N. tabacum* genes used in this work.

Primer names	Primer Sequences (5'–3')
attB1SDK-14-3-3D-FW	gca ggc ttc gaa gga gat aga acc ATG GCC GTA CCG GAA AAT TTA AC
attB2-14-3-3D-RV	aag ctg ggt cTC AAG CCT CGT CCA TCT GC
attB2-14-3-3Dss-RV	aag ctg ggt cAG CCT CGT CCA TCT GCT CC
14–3-3A-FW	gca ggc ttc gaa gga gat aga acc ATG GCA TCG CCG CGC GAG
14–3-3A-RV	aag ctg ggt cTT ACT GCT GCT CCT CCG CTT
14–3-3Ass-RV	aag ctg ggt cCT GCT GCT CCT CCG CTT TT
SCI1(Ct11)Mut1NewFW	G AGG AAG CAT AAG AGA *GC*T *G*CG CCT *G*CT *G*CT CCA CGA GAT GAA G
SCI1(Ct11)Mut1NewRV	C TTC ATC TCG TGG AG*C* AG*C* AGG CG*C* A*GC* TCT CTT ATG CTT CCT C
SCI1mut2-FW	G AAG GAG AAG CAC AAA *G*CC CAT *G*CT *GC*T GAA GAG AAG AAG TCA GG
SCI1mut2-RV	CC TGA CTT CTT CTC TTC A*GC* AG*C* ATG GG*C* TTT GTG CTT CTC CTT C
attB1-NtWee1-FW	gca ggc ttc gaa gga gat aga acc ATG AAG AGG AAA ACC CTA AAT C
attB2-NtWee1-RV	aag ctg ggt cTT ACT TGT TAG CAT TTC TTT G
BP1	ggg gac aag ttt gta caa aaa agc agg ctt c
BP2	ggc gac cac ttt gta caa gaa agc tgg gtc

Capital letters indicate the gene-specific sequences of each primer. Small letters are part of the attB1 and attB2 recombination sites. Underlined and italic letters show the mutations introduced in the SCI1 sequence.

### *In vitro* Pulldown

Recombinant proteins HIS-14-3-3D2, GST-SCI1, and GST alone were expressed in *E. coli* BL21(DE3) Rosetta cells after induction with 0.1 mM IPTG for 2 h. Total soluble proteins were extracted with PBS Buffer [140 mM KCl, 10 mM Na_2_HPO_4_, 1.8 mM KH_2_PO_4_, 1 mM PMSF, 50 μg/ml lysozyme, and 1% protease inhibitor cocktail for general use (Roche), pH 7.3], sonication, and centrifugation. Soluble proteins from the indicated extracts were mixed and incubated overnight at 4°C with equilibrated 100% Glutathione Sepharose 4 FastFlow bead slurry (GE Healthcare). Beads were washed three times with PBS supplemented with 1 mM PMSF. Bound proteins were eluted with 100 μl of 2x Laemmli Sample Buffer and boiled. The different fractions were resolved in 12% bis-acrylamide gel, immunoblotted with anti-GST (Sigma SAB4200055), or anti-HIS primary antibody (Sigma H1029), and visualized by enhanced chemiluminescence reaction (ECL) reaction.

### Co-immunoprecipitation

Soluble proteins were obtained from *N. tabacum* 35S_prom_::SCI1-GFP stigmas/styles in extraction buffer (50 mM Tris-HCl pH7.5, 75 mM NaCl, 1% Triton X-100, 5% glycerol, 2 mM EDTA, 5 mM Na_3_VO_4_, 5 mM NaF e 20 mM β-glycerolphosphate, and cOmplete™, EDTA-free Protease Inhibitor Cocktail—Roche). The material was vortexed for 10 min on ice, submitted to sonication (six pulses of 30 s) at 30% Branson Sonifier™ (S-450 digital ultrasonic cell disruptor/homogenizer, Fisher Scientific), and centrifuged at 18,500 × *g* for 20 min, and the supernatant was collected. HIS-14-3-3D2 protein was expressed on *E. coli* BL21(DE3) Rosetta strain and extracted with sonication (five pulses of 30 s) in lysis buffer [Tris-HCl 50 mM pH 7.4, NaCl 75 mM, 1 mM Protease Inhibitor Cocktail for General Use (Sigma), and 50 μg/ml lysozyme]. Both soluble protein extracts were mixed and incubated with monoclonal anti-GFP antibody (Sigma G6539) at 4°C for 2 h, under gentle agitation. Then, Protein G Sepharose beads four-fast flow (GE Healthcare) was added, followed by incubation at 4°C for 2 h and centrifugation. The precipitated immunocomplexes were washed six times with extraction buffer, eluted from the beads by boiling in SDS-PAGE sample buffer, and resolved on 12.5% acrylamide gels. Immunoblots were carried out using anti-HIS primary antibody (Sigma H1029) or anti-HIS-SCI1-polyclonal antibody (produced in chicken by IgY Biotech—Brazil, against the recombinant HIS-SCI1 protein produced in *E. coli*).

### Subcellular Localization and Bimolecular Fluorescence Complementation in *Nicotiana benthamiana* Leaves

The recombinant plasmids (35S_prom_::SCI1-GFP and the nuclear markers AtFibrillarin-mRFP and AtCoilin-mRFP—Koroleva et al., 2009) were transformed into *A. tumefaciens* strain C58C1(pGV2260) and infiltrated on leaves of 5–6 weeks old *N. benthamiana* plants, as previously described (Bracha et al., 2002). Fluorescence detection was observed 24–72 h following infiltration. Cells were stained with DAPI (4′,6-diamidino-2-phenylindole) and examined under a Leica TCS SP5 confocal laser scanning microscope (Leica Microsystems—Germany). GFP excitation was done with an argon laser at 488 nm, and the spectral detector was set between 500 and 550 nm, while RFP excitation was done with a laser at 543 nm and a spectral detector set between 630 and 680 nm. The fluorescence of DAPI was obtained with excitation at 340–380 nm and captured at 425–450 nm. Image analysis was carried out with Leica LCS, ImageJ, and Illustrator CS6.

To verify interaction partners *in planta*, the coding sequences of SCI1, 14-3-3A1 and 14-3-3D2 were subcloned into pK7m34GW or pH7m34GW vectors with nGFP or cGFP fragments, under the control of 35S promoter (Karimi et al., 2007). These split-GFP Gateway-based vectors were infiltrated in *N. benthamiana* leaves and used for BiFC analysis, as Bracha-Drori et al. (2004) described.

### Stable Transgenic BY-2 Cell Lines

*Nicotiana tabacum* BY-2 cells were cultivated and stably transformed, as described by Nagata et al. (1992) and Nocarova and Fischer (2009), with constructions 35S_prom_::SCI1-GFP and 35S_prom_::SCI1mut3-GFP. Samples were stained with Hoechst 33342 (Sigma) or DAPI, analyzed, and photographed at confocal microscope Leica TCS SP5. Treatment with MG132 (50 μM in DMSO) was used to block the proteolytic activity of the 26S proteasome complex. The fluorescence intensity emitted by SCI1-GFP, SCI1mut3-GFP, and DAPI was quantified, and graphs were obtained using the ImageJ software histogram tool.

#### Accession Numbers

SCI1 (GQ272329), 14-3-3D2AS (AB119473), 14-3-3A1 (AB119466), 14-3-3G1 (AB119477), 14-3-3H1 (AB119478), SKP1 (JN793550), CSN6a (XM002267120), BTB/POZ (XP016450235), and Wee1 (AM408785).

## Results

### SCI1 Interacts With 14-3-3D From the Non-Epsilon Group

**Figure 1:**
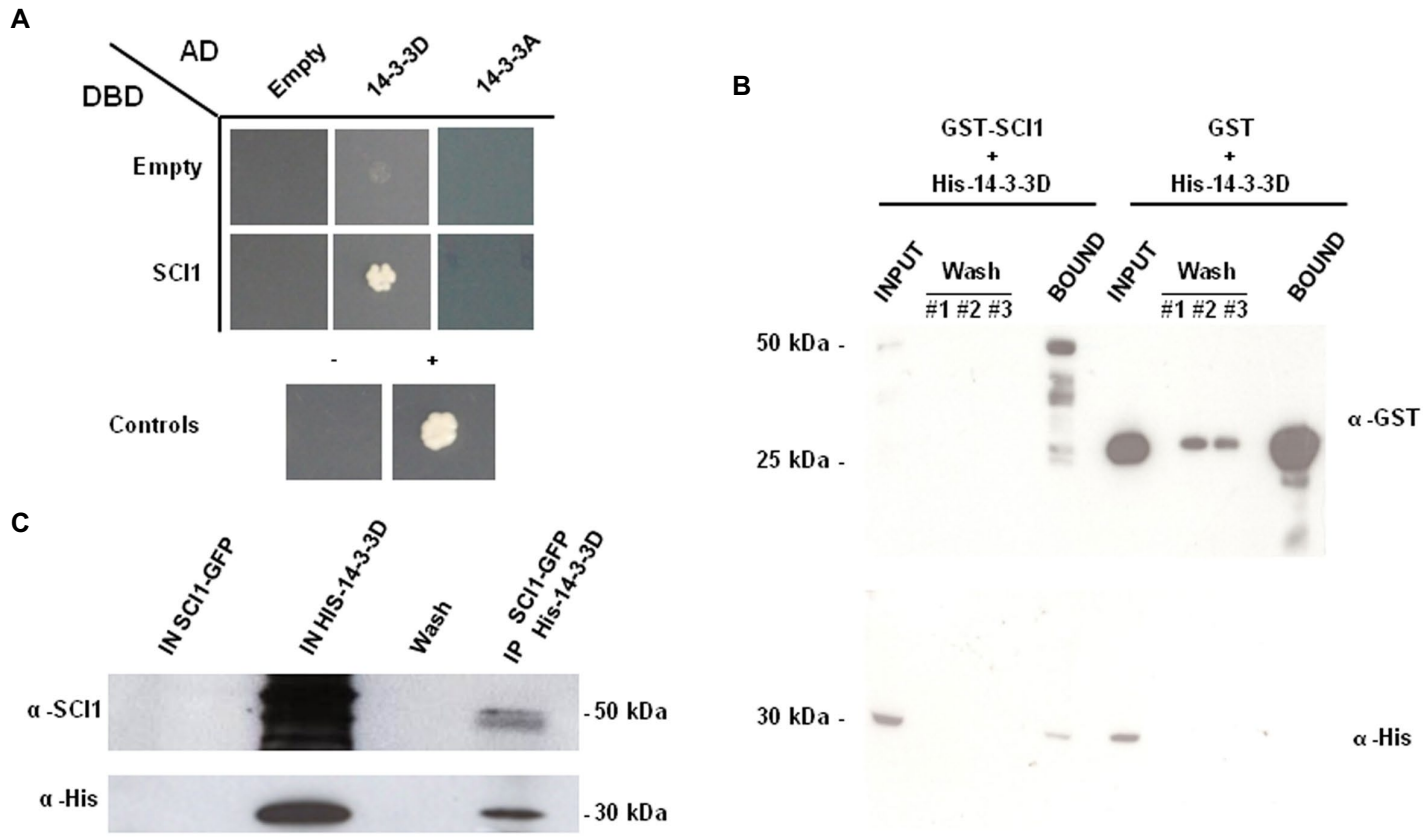
**(A)** Yeast two-hybrid (Y2H) binary assays of the interaction of BD-SCI1 with AD-14-3-3D and AD-14-3-3A proteins. Yeast PJ69-4a cells with empty vectors are unable to grow on a selective medium without histidine. The self-activation of BD-SCI1, AD-14-3-3D, and AD-14-3-3A were negative. Cells could only grow when BD-SCI1 and AD-14-3-3D proteins were present, showing HIS3 reporter gene expression and confirming the interaction between these proteins. **(B)** *In vitro* pulldown with recombinant GST-SCI1, GST, and HIS-14-3-3D produced in *Escherichia coli*. **(C)** Co-immunoprecipitation of HIS-14-3-3D protein using an extract of stigmas/styles from *Nicotiana tabacum* 35S_prom_::SCI1-GFP plants. The smear observed at the input HIS-14-3-3D lane is due to background recognition of *E. coli* proteins by the anti-HIS-SCI1 polyclonal antibody.

To study the molecular mechanisms underlying the control of cell proliferation by SCI1, we constructed a tobacco stigma/style cDNA library through the Y2H system and screened it for SCI1-interacting proteins. The entire SCI1 coding sequence was used as bait (BD-SCI1), and approximately 1 × 10^6^ virtual transformants were screened. Among the yeast colonies able to grow on the selective medium lacking histidine (His), we identified two clones encoding 14-3-3D2 and one encoding the 14-3-3A1 protein, both from the Non-Epsilon group (Supplementary Figure S3). Consistently, three putative 14-3-3 mode I (RSX-pS/pT-XP) binding motifs were found by MotifScan software (Pagni et al., 2004) on the SCI1 amino acid sequence (Supplementary Figure S2). After verification by Y2H binary assay, the interaction between SCI1 and 14-3-3D2 was confirmed, but not between SCI1 and 14-3-3A1 (Figure 1A). For simplicity, from this point on, these sequences will be referred to as 14-3-3A and 14-3-3D.

The interaction between SCI1 and 14-3-3D was further verified by an *in vitro* pulldown assay (Figure 1B). The binding of HIS-14-3-3D to glutathione beads in the presence of GST-SCI1, but not of GST alone, showed the specific interaction of 14-3-3D with SCI1 (Figure 1B—bottom panel). We also assayed the interaction of SCI1 and 14-3-3D by immunoprecipitation of stigma/style proteins from a transgenic tobacco plant expressing 35S_prom_::SCI1-GFP mixed with an extract of *E. coli* expressing HIS-14-3-3D. A band corresponding to the expected size of HIS-14-3-3D (33.6 kDa) was detected, confirming its interaction with SCI1 (Figure 1C). Of note, two bands were revealed with the anti-SCI1 antibody, consistent with SCI1 protein being modified, possibly phosphorylated by the stigma/style proteins as proposed previously (DePaoli et al., 2011). Altogether, these results confirm the interaction between SCI1 and 14-3-3D.

### 14-3-3A and 14-3-3D Form Homo- and Heterodimers in the Cytoplasm of Cells in Interphase

Expression analysis shows that both 14-3-3A and 14-3-3D (Supplementary Figure S4) are well expressed in different tobacco organs (roots, stems, young leaves, mature leaves, young flowers, young stigmas/styles, mature flowers, and dry capsules). Meanwhile, some other 14-3-3 isoforms (14-3-3E and 14-3-3F) have low expression levels (Supplementary Figure S4). It is worth mentioning that 14-3-3A and 14-3-3D are not the 14-3-3 proteins with the highest expression levels in stigmas/styles (Supplementary Figure S5), the organs used to construct the Y2H cDNA library.

**Figure 2:**
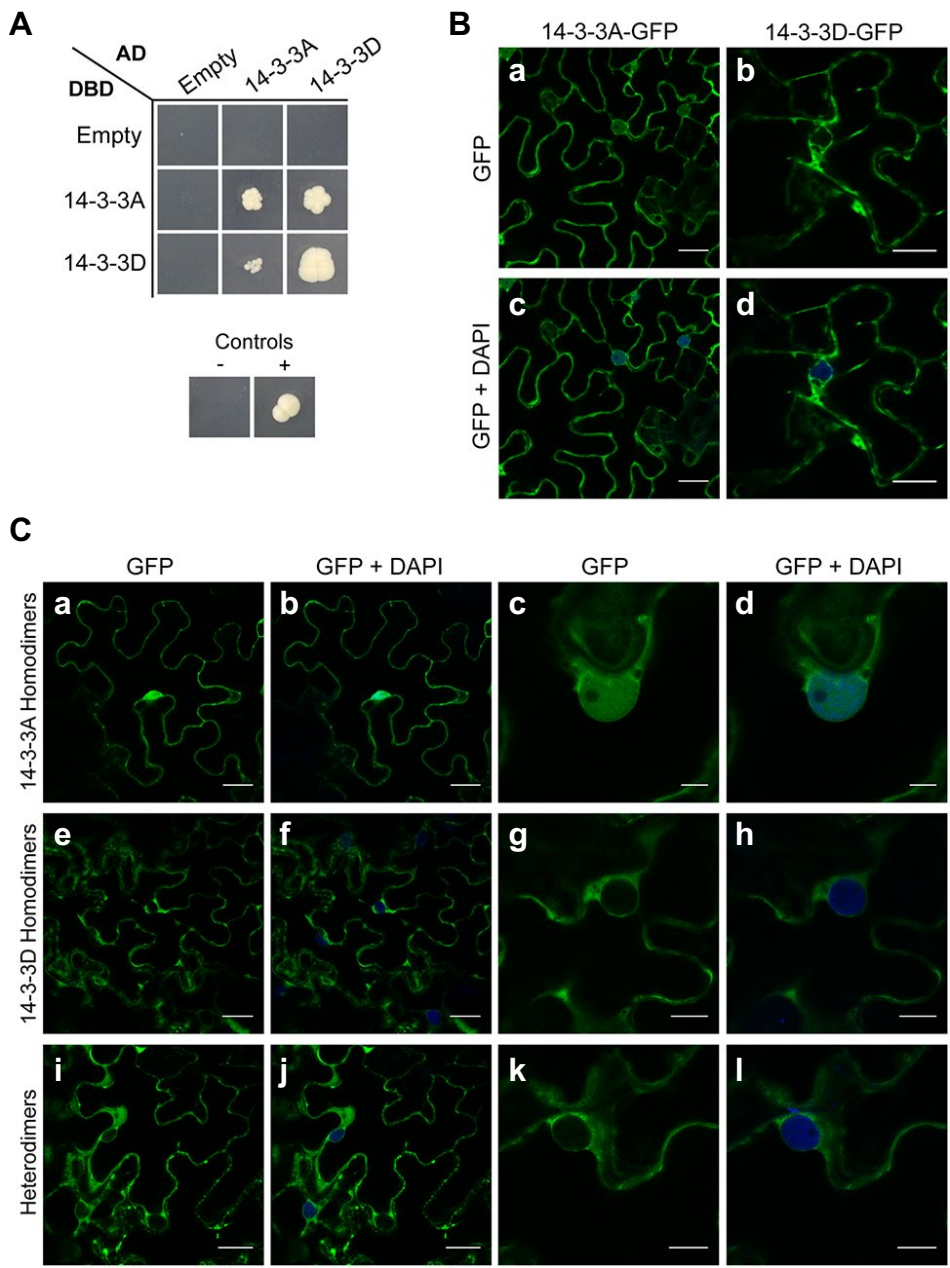
Dimerization and subcellular localization of 14-3-3A and 14-3-3D. **(A)** Y2H assay testing the dimerization of 14-3-3A and 14-3-3D proteins. Selective medium: SC-Leu-Trp-His +1 mM 3AT. C−: Negative interaction control; C+: positive interaction control. AD—Activation domain; DBD—DNA binding domain. **(B)** Subcellular localization of 14-3-3A-GFP and 14-3-3D-GFP. **(Ba)** Cytosolic and nuclear localization of 14-3-3A-GFP, bar 20 μm. **(Bc)** Merged image of 14-3-3A-GFP localization and nuclear DAPI staining, bar 20 μm. **(Bb)** Cytosolic localization of 14-3-3D-GFP, bar 15 μm. **(Bd)** Merged image of 14-3-3D-GFP localization and nuclear DAPI staining, bar 15 μm. **(C)** Bimolecular Fluorescence Complementation (BiFC) assay showing the dimerization of 14-3-3A and 14-3-3D. **(Ca,D)** 14-3-3A-nGFP × 14-3-3A-cGFP, showing 14-3-3A homodimers formation in cytosol and nucleus, bars 20 and 5 μm. **(Cb,D)** Merged images of 14-3-3A homodimers and DAPI staining, bars 20 and 5 μm. **(Ce,G)** 14-3-3D-nGFP × 14-3-3D-cGFP, showing 14-3-3D homodimers formation in cytosol, bars 20 and 10 μm. **(Cf,H)** Merged images of 14-3-3D homodimers and DAPI staining, bars 20 and 10 μm. **(Ci,K)** 14-3-3A-nGFP × 14-3-3D-cGFP, showing heterodimers formation in cytosol, bars 40 and 10 μm. **(Cj,L)** Merged images of heterodimers and DAPI staining. Subcellular localization and BiFC experiments were performed as transient expressions in *N. benthamiana* leaves.

Although 14-3-3A interaction with SCI1 was not confirmed, we chose to investigate whether 14-3-3A and 14-3-3D can form homo and/or heterodimers, taking 14-3-3A as a representative member of the distant and larger subclade within the 14-3-3 family (Supplementary Figure S3). Therefore, we performed Y2H and BiFC assay with split GFP. For the Y2H, yeast cells were co-transformed with combinations of the four constructions (AD-14-3-3A; BD-14-3-3A; AD-14-3-3D; and BD-14-3-3D) and assayed for their ability to grow on the selective medium lacking histidine. The Y2H results have demonstrated the formation of homodimers of 14-3-3A, homodimers of 14-3-3D, and the heterodimers of 14-3-3A and 14-3-3D (Figure 2A).

Transient expression of 14-3-3A-GFP in *N. benthamiana* leaves demonstrated its localization in both nucleus and cytoplasm (Figures 2Ba,c), while 14-3-3D-GFP is cytoplasm restricted (Figures 2Bb,d). BiFC assays confirmed the *in planta* formation of homo- and heterodimers and established their subcellular localization. 14-3-3A homodimers were detected in both the nucleus and cytoplasm (Figures 2Ca**–**d), whereas 14-3-3D homodimers (Figures 2Ce**–**h) and the 14-3-3D/14-3-3A heterodimers (Figures 2Ci**–**l) were found only in the cytoplasm.

### SCI1 Interacts With the 14-3-3D Protein in the Nucleus in Early Mitosis

**Figure 3:**
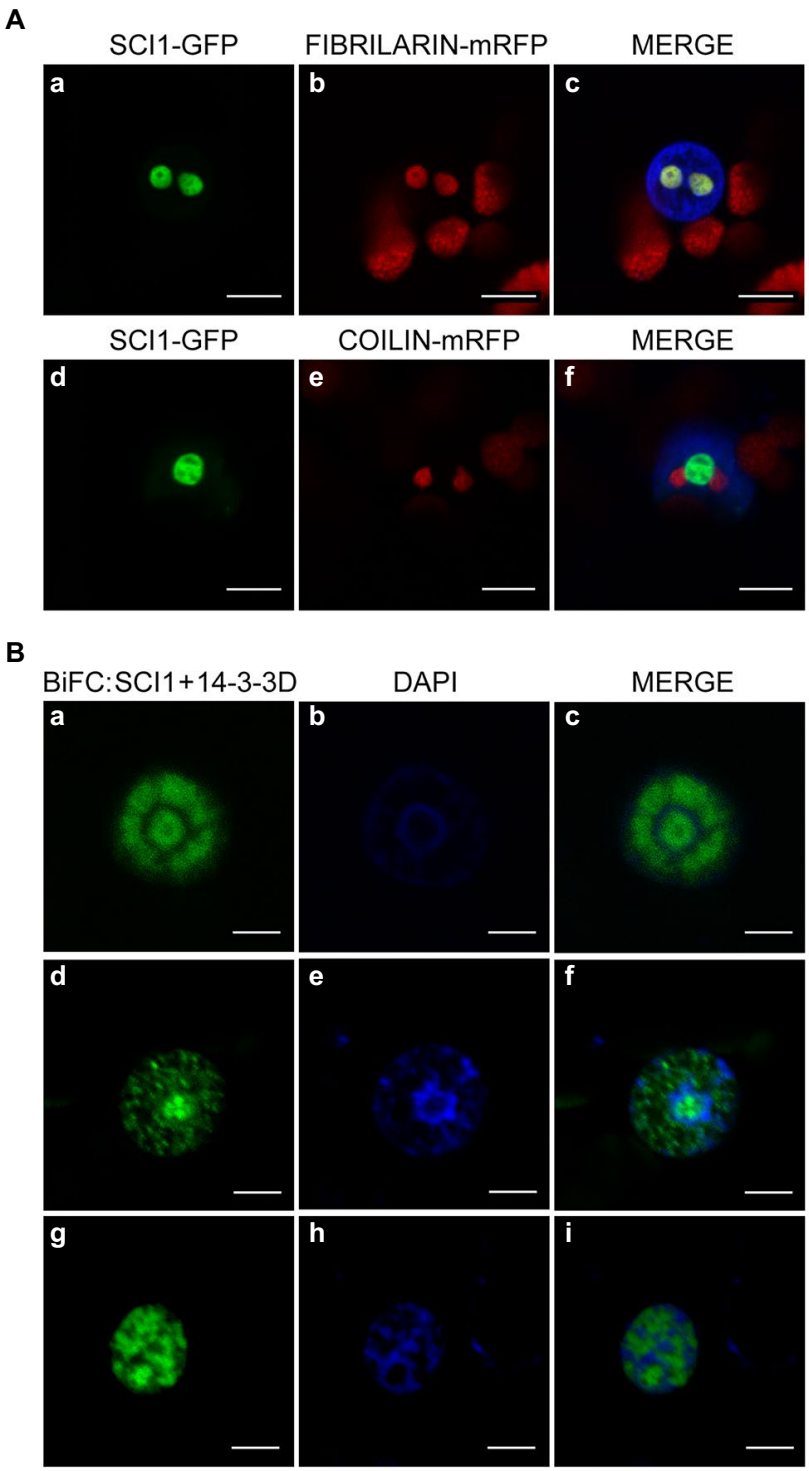
**(A)** Co-localization of SCI1-GFP with the nuclear markers AtFibrillarin-mRFP for nucleoli and AtCoilin-mRFP for Cajal bodies (Koroleva et al., 2009), in transiently transformed *N. benthamiana* leaf cells. The red color organelles observed outside of the nucleus correspond to chloroplasts (auto-fluorescence). **(B)** BiFC assays indicating that SCI1 interacts *in vivo* with 14-3-3D protein in the nucleus of epidermal cells of *N. benthamiana* leaves. This interaction was observed only in cells at G2/prophase (early mitosis). Three different cells are shown in each line **(Ba,d,g)**. Blue indicates DAPI stained DNA. Green indicates the interaction of SCI1-nGFP and 14-3-3D-cGFP **(a–i)**. nGFP, N-terminal fragment of GFP (amino-acid residues 1–149); cGFP, C-terminal GFP fragment (amino-acid residues 150–238). Scale bars—5 μm.

Transient expression of SCI1-GFP in *N. benthamiana* leaves shows it is localized in the nucleus (DePaoli et al., 2011, 2014), while 14-3-3D was found only in the cytoplasm (above), and yet we have demonstrated their interaction (Figure 1). We ruled out possible mislocalization of SCI1-GFP due to lack/need of tissue-specific factors in leaves by confirming that SCI1-GFP has the same subcellular localization in both stigma/style cells of *SCI1prom::SCI1-GFP* transgenic plants (Cruz et al., 2021) and cells of transiently transformed leaves of *N. tabacum* and *N. benthamiana*. Furthermore, SCI1-GFP co-localizes with fibrillarin-RFP (nucleoli marker) and not with coilin-RFP (Cajal bodies marker), demonstrating that it is contained in the nucleolus (Figure 3A).

Our previous approaches evaluated the interaction between SCI1 and 14-3-3D by either heterologous or cell disruptive methods; then, we performed subsequent interaction analysis in intact plant cells. BiFC assays, performed in *N. benthamiana* leaves, revealed that SCI1 and 14-3-3D interact in the nucleus (Figure 3B), more specifically, in areas interspersed with early-condensing chromatin (Figures 3Ba**–**i), which occurs at the transition G2/prophase as evaluated by the DAPI staining (Li et al., 2005; San Martin et al., unpublished results).

### SCI1 Is Degraded by the 26S Proteasome During Cell Division

**Figure 4:**
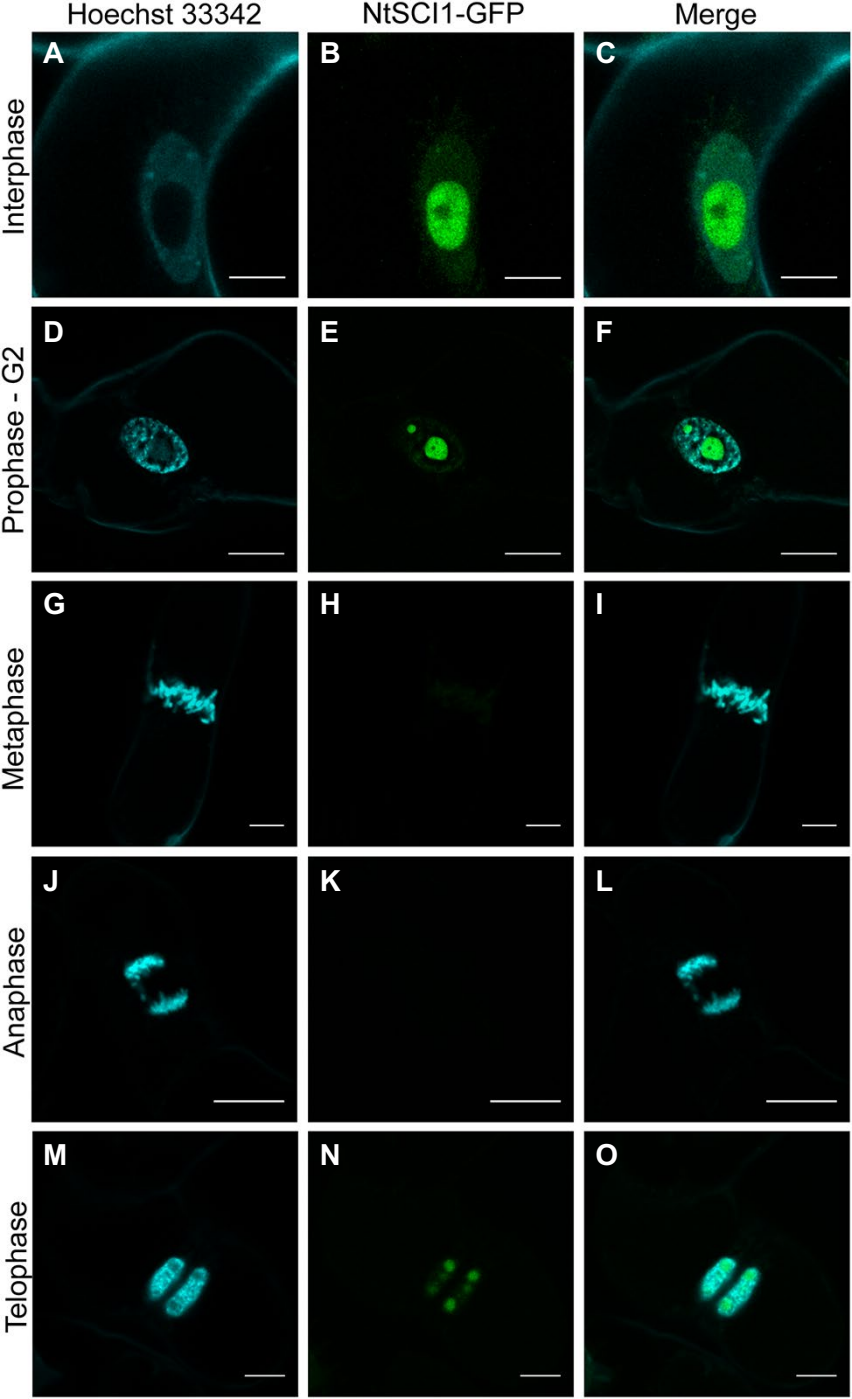
Stigma/Style Cell-Cycle Inhibitor 1 (SCI1) dynamics throughout cell-cycle progression. Confocal microscopy images showing SCI1-GFP subcellular localization in each cell-cycle phase. Tobacco BY-2 cells stably transformed with 35S_prom_::SCI1-GFP were stained with Hoechst 33342. Images were captured using Leica TCS SP5 (Leica Microsystems). Scale bars—10 μm **(A–C)** and 20 μm **(D–O)**.

**Figure 5:**
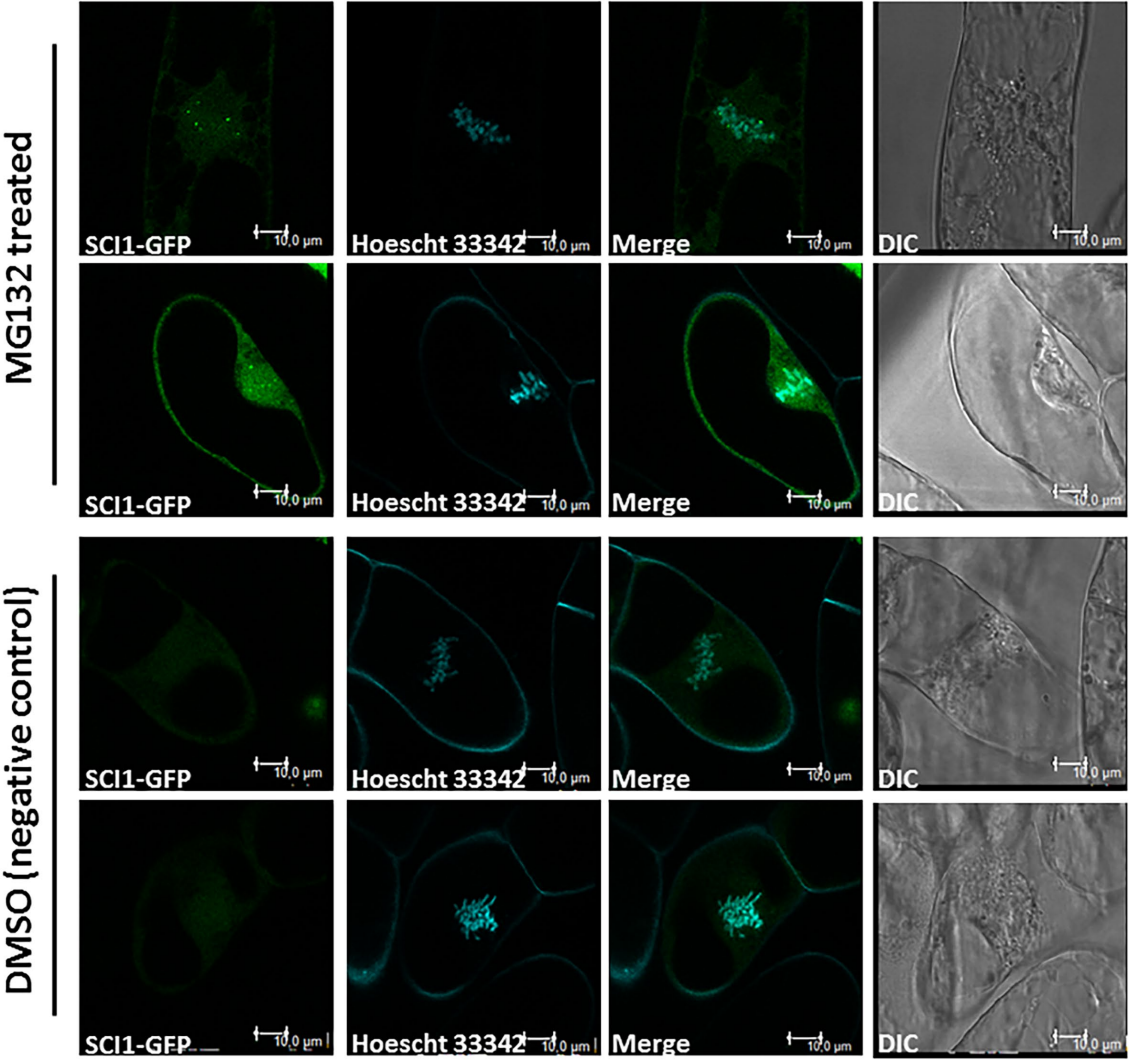
Stigma/Style Cell-Cycle Inhibitor 1 is stabilized upon treatment with MG132, an inhibitor of 26S proteasome. Confocal microscopy images showing SCI1-GFP in metaphase cells after treatment with MG132 (50 μM in DMSO) and control (DMSO). Tobacco BY-2 cells stably transformed with 35S_prom_::SCI1-GFP were stained with Hoechst 33342. Images were captured using Leica TCS SP5 (Leica Microsystems). DIC—Differential interference contrast microscopy. Scale bars—10 μm.

To explore SCI1 protein behavior during cell-cycle progression, *N. tabacum* BY-2 cells were stably transformed to express *35S_prom_::SCI1-GFP*. During interphase and prophase, the chimeric protein was clearly observed in nucleoli (Figures 4A–F). However, at metaphase, SCI1-GFP was hardly visible (Figures 4G–I), and it was no longer detected at anaphase (Figures 4J–L; see also Supplementary Figure S6). At the end of mitosis (telophase), SCI1-GFP became visible again into the reassembling nascent nucleoli (Figures 4M–O). To test the hypothesis that SCI1 is degraded at early mitosis by the 26S proteasome pathway, an important mechanism to regulate cell-cycle phase transitions (Genschik et al., 2014), we treated the tobacco BY-2 cells stably expressing SCI1-GFP with the 26S proteasome inhibitor MG132. This treatment significantly minimized SCI1 degradation during cell division, which is detected spread on the cell after nuclear envelope breakdown (Figure 5). Notably, under MG132 treatment, SCI1 protein also accumulated in several specific positions of the condensed metaphase chromosomes. A detailed analysis was performed in different MG132 treated cells, revealing that the number and position of the SCI1-spots on the chromosomes (Supplementary Figure S7) are consistent with SCI1 being associated with the tobacco nucleolar organizer regions—NOR (Parokonny and Kenton, 1995).

Altogether, these data indicate that SCI1 interacts with 14-3-3D in the nucleus, is ultimately degraded at a specific time during cell division (late prophase/metaphase), preceding the separation of chromosomes, and reappears at telophase. Thus, SCI1 stability is dependent on the cell-cycle phase, showing similar behavior to other cell-cycle regulators (Genschik et al., 2014; Koepp, 2014), further supporting our claim of SCI1 as a molecular regulator of cell proliferation.

### SCI1 Degradation by the 26S Proteasome Is Dependent on Serine Residues

**Figure 6:**
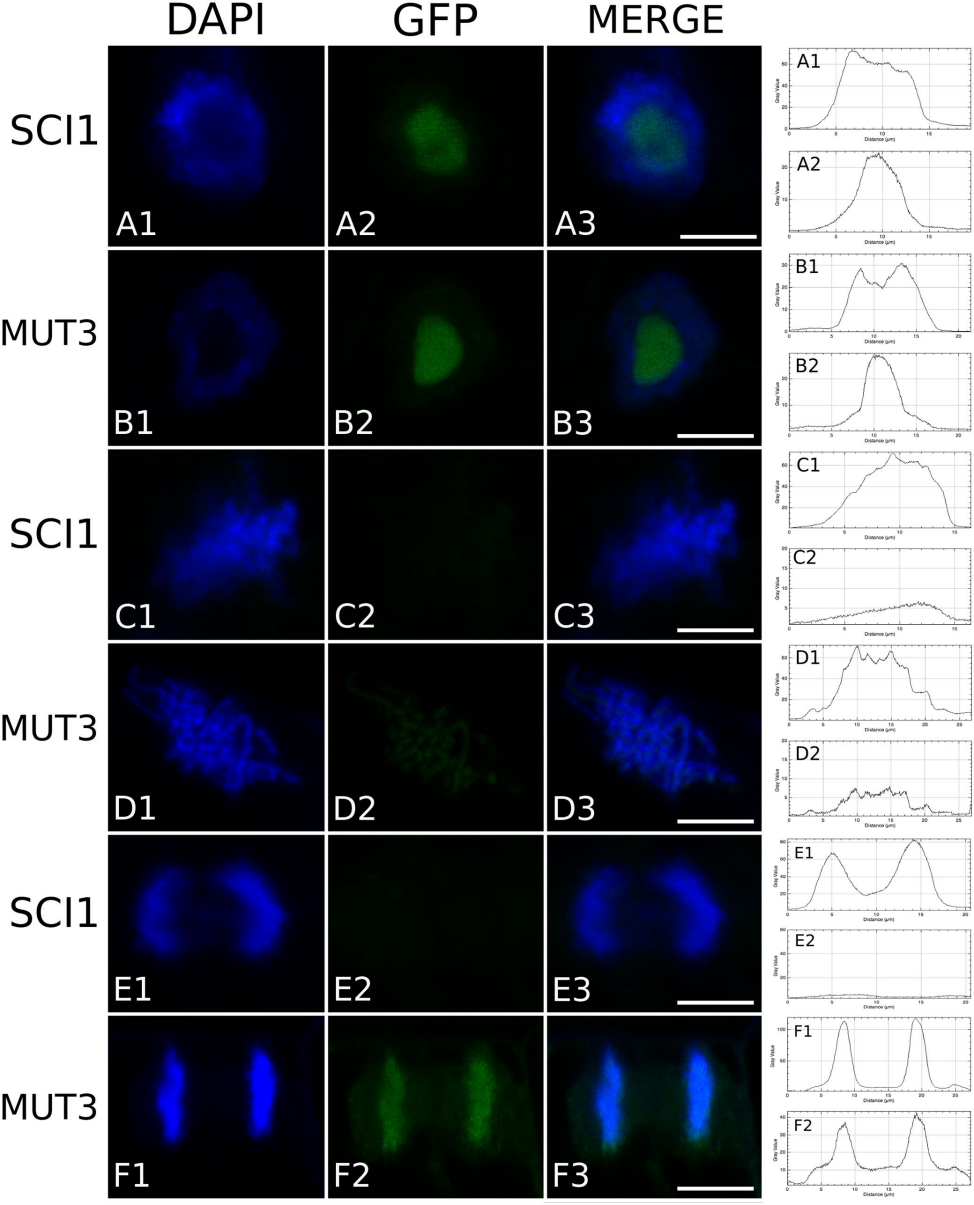
A mutated version of SCI1 lacking seven serine residues is not degraded during cell division. Confocal microscopy images showing SCI1-GFP and SCI1mut3-GFP in different cell-cycle phases: interphase, metaphase, and anaphase. Tobacco BY-2 cells stably transformed with 35S_prom_::SCI1-GFP and 35S_prom_::SCI1mut3-GFP were stained with DAPI. Images were captured using Leica TCS SP5 (Leica Microsystems). Identical settings were used to compare fluorescence intensity, which was quantified and is shown on the graphics at right (two graphics for each line of cells). The first graphic (above) refers to the DAPI image’s fluorescence, while the second graphic (below) represents the GFP fluorescence. Scale bars 7.5 μm.

To further investigate SCI1 stability and cellular behavior, we mutated seven conserved serine residues of SCI1 (S17, 18, 20, 21, 54, 56, and 57) to alanine (SCI1mut3), which lay within the predicted 14-3-3 binding sites in the N-terminal region (as shown in Supplementary Figure S2). Then, SCI1mut3 was fused to GFP, and the chimeric gene construction was introduced into BY-2 cells. Stably transformed BY-2 cells demonstrated that, at interphase, SCI1mut3-GFP and SCI1-GFP have the same localization, in the nucleoli. On the other hand, at cell division, while SCI1-GFP is degraded before metaphase and still undetected in anaphase, SCI1mut3-GFP is stable at these different phases (Figure 6; note fluorescence quantification and comparison for SCI1-GFP and SCI1mut3-GFP). Notably, SCI1mut3-GFP is associated with chromosomes at metaphase (Figure 6 and Supplementary Figures S8, S9). Therefore, SCI1 proteasomal degradation during mitosis may rely on the (phospho)-serine residues present at the putative 14-3-3 binding sites. Additionally, we identified different proteins related to the ubiquitin-proteasome pathway using 14-3-3D as a bait on a new Y2H screening: CSN6A, a subunit of the COP9 signalosome (homologous to At5g56280); a BTB/POZ domain-containing protein (homolog of At1g03010); and SKP1, a core component of the SCF family of E3 ubiquitin ligases (homologous to At1g75950). Furthermore, 14-3-3D, 14-3-3G, and 14-3-3H were found in the screening as interaction partners of 14-3-3D, which was confirmed by a binary Y2H assay (Supplementary Figure S10). The interaction of 14-3-3D with components mediating proteasomal-dependent degradation supports the role of 14-3-3D as a docking degron for SCI1 in mitosis.

## Discussion

### SCI1 Interacts With a Specific 14-3-3 Protein

The Y2H screening used to search for SCI1-interacting proteins has identified only 14-3-3A and 14-3-3D and no other 14-3-3 protein. As Y2H screenings are hardly exhaustive, we cannot ruled out the possibility that SCI1 interacts with other 14-3-3 proteins expressed in stigmas/styles (Supplementary Figure S5). On the other hand, 14-3-3C1 has the highest expression in these organs and should be well represented among the clones available at the Y2H library. Nonetheless, no clone encoding 14-3-3C1 was found in the screening, suggesting specificity in the interaction of SCI1 with members of the 14-3-3 family.

We could not confirm the interaction between SCI1 and 14-3-3A in the Y2H binary assay (Figure 1A). If 14-3-3A interacts with SCI1, it may depend on additional factors or protein modifications that rarely occur in yeast or are challenging to be detected. The screening of the stigma/style Y2H cDNA library using 14-3-3D as bait has identified only 14-3-3D, 14-3-3G, and 14-3-3H as its partners within the 14-3-3 family, despite the expression of other 14-3-3s (Supplementary Figure S5). Although 14-3-3D may form a heterodimer with 14-3-3G and 14-3-3H (Supplementary Figure S10), it is unlikely that these other 14-3-3 proteins directly interact with SCI1 as homodimers, since they were not recovered during the screening using SCI1 as bait. Conversely, heterodimers of 14-3-3D may be important for SCI1 regulation and were not thoroughly investigated here. Nevertheless, it seems that a functional specialization may exist among the 14-3-3 proteins, in which not all heterodimers are formed or are functionally interchangeable, as already observed in other plant systems (Paul et al., 2012; Pallucca et al., 2014). Future experiments may shed some light on the patterns of dimerization that actually occur *in vivo* to regulate SCI1.

14-3-3A and 14-3-3H belong to the same phylogenetic subgroup, while 14-3-3D and 14-3-3G are placed together in another subgroup within the Non-Epsilon group (Supplementary Figure S3). The interactions of 14-3-3D found in our Y2H screening were restricted to these two phylogenetic branches. Similarly, the 14-3-3s from other branches were not identified as SCI1 interactors in this study. Such a 14-3-3 interaction specificity is not always the case in plants. For example, several Arabidopsis 14-3-3 proteins (14-3-3 Lambda, Kappa, Epsilon, Phi, and Omega) interact with the transcription factors BZR1 (BRASSINAZOLE-RESISTANT 1) and BES1 (BRI1-EMS-SUPPRESSOR 1) from the brassinosteroid signaling pathway (de Boer et al., 2012).

### SCI1 Proteasomal Degradation Occurs in Early Mitosis and Is Possibly Mediated by 14-3-3D

Stigma/style cell-cycle inhibitor 1 mutated in seven serines (SCI1mut3; Supplementary Figure S2) is not degraded during mitosis, as it occurs for the non-mutated SCI1 protein (Figure 6; Supplementary Figures S8, S9). Interestingly, SCI1mut3-GFP is associated with the condensed chromatin and with microtubules during cell division (metaphase and anaphase), localizations never observed for the non-mutated version. At these cell division stages, SCI1mut3-GFP is spread all over the entire length of the chromosomes (Figure 6; Supplementary Figures S8, S9). On the other hand, SCI1-GFP in cells treated with MG132 concentrates on chromosomes’ specific spots (Figure 5; Supplementary Figure S7), besides being dispersed throughout the cell. The different behaviors observed may be explained by the fact that proteasome inhibition by MG132 does not affect SCI1 only, but many other proteins that should be degraded during cell division.

Previous studies in other systems have demonstrated the importance of 14-3-3 proteins as signaling integrators for cell-cycle control (Peng et al., 1997; Laronga et al., 2000; van Hemert et al., 2001; Gardino and Yaffe, 2011). In plants, some of the Arabidopsis Non-Epsilon 14-3-3 proteins can bind to the CDC25 phosphatase, as well as to rescue cell-cycle defects in fission yeast mutants (Sorrell et al., 2003) and to contribute to the robustness of the DNA damage and spindle checkpoints in budding yeast (Usui and Petrini, 2007; Grandin and Charbonneau, 2008; Engels et al., 2011; Caydasi et al., 2014; Bokros et al., 2016; Gryaznova et al., 2016; Chappidi et al., 2019). Additionally, Non-Epsilon Arabidopsis 14-3-3 proteins were shown to interact with Wee1, a kinase that inhibits the cell-cycle in the nucleus of interphase cells (Grønlund et al., 2009 and references therein). Wee1 degradation has been observed in yeast and animals occurring in G2/M and is important for mitotic progression (Owens et al., 2010). Indeed, 14-3-3 proteins have been shown to interact with Wee1 in yeast and animals (Honda et al., 1997; Wang et al., 2000) and regulate its stability and kinase activity in G2/M (Wang et al., 2000). Interestingly, 14-3-3D, but not 14-3-3A, can interact with the tobacco Wee1 (Supplementary Figure S11), supporting a role for 14-3-3D in the cell division signaling pathway.

**Figure 7:**
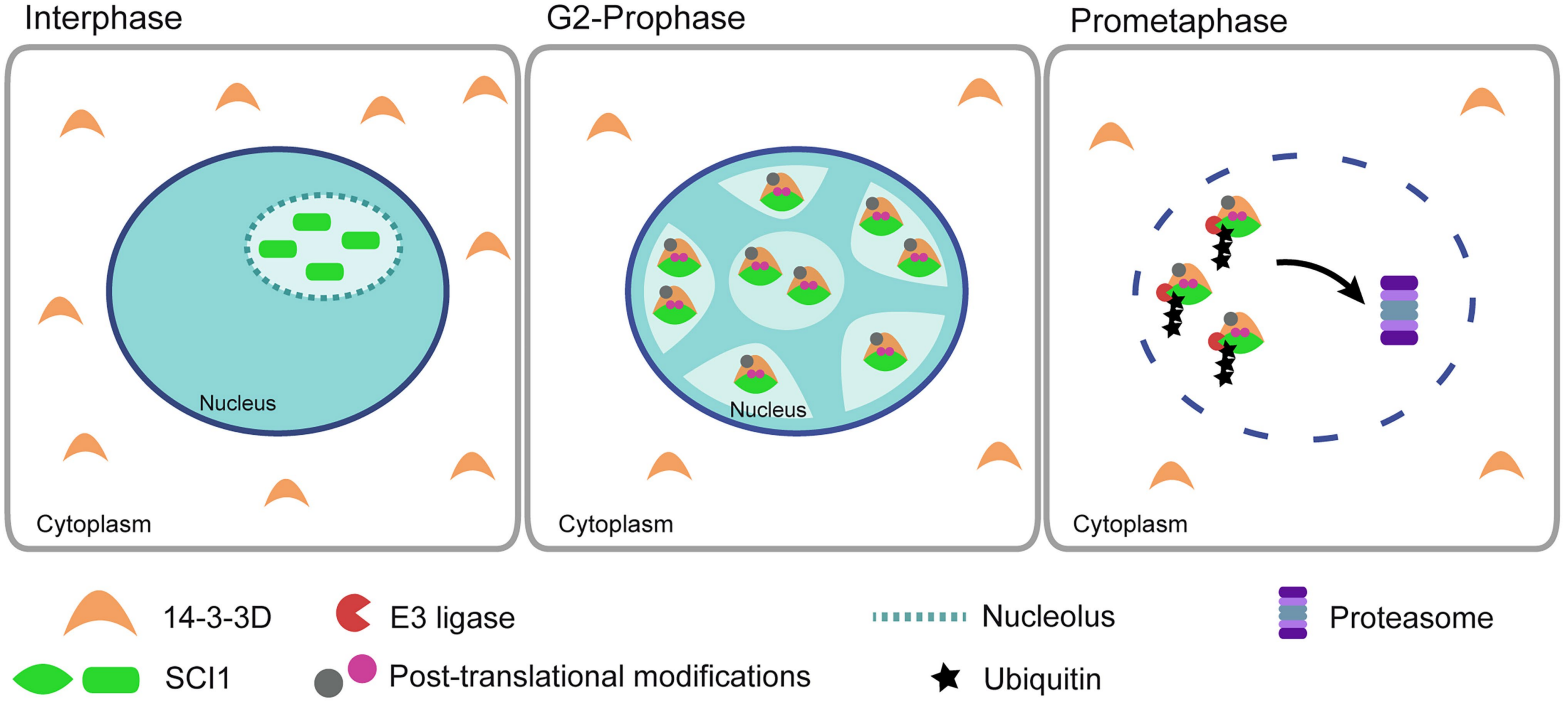
A proposed model illustrates the interaction of SCI1 and 14-3-3D during early mitosis. During interphase (DAPI staining, in blue, is well spread in the nucleus), SCI1 is in the nucleolus, and 14-3-3D is in the cytoplasm. Signals to start cell division promote the movement of 14-3-3D to the nucleus. In parallel, cell division signals cause SCI1 post-translational modifications (possibly phosphorylation by a still unknown kinase). At G2/prophase, chromatin is starting its condensation (shown in blue), and SCI1 and 14-3-3D interact at the nucleoplasm. At prometaphase, 14-3-3D mediates SCI1 26S proteasome degradation, which allows cell division to progress.

It is known that 14-3-3 proteins may act as adaptors or “chaperone molecules” that can move freely from cytoplasm to the nucleus and *vice-versa* (Mhawech, 2005). During interphase, 14-3-3D is cytoplasm localized (Figure 2) and moves to the nucleus in early mitosis, where and when it interacts with SCI1 (Figure 3). It is possible that the nuclear shuttling of 14-3-3D is influenced by PTMs in its own amino acid sequence. PTMs, such as phosphorylation, have already been identified in 14-3-3 proteins (Wilson et al., 2016). Therefore, we suggest that cell division signaling promotes 14-3-3D PTM(s) in the cytoplasm and, consequently, modifies its subcellular localization. Alternatively, cell division signaling could promote PTM(s) of other protein(s), which would then interact with 14-3-3D in the cytoplasm and translocate it to the nucleus, as already described to some Arabidopsis 14-3-3 proteins (Paul et al., 2005). In the nucleus, the interaction between SCI1 and 14-3-3D could be facilitated by PTM(s) on SCI1 (Figure 7), most likely by phosphorylation. The presence of SCI1 differentially migrating bands in the 1D western blot of the co-immunoprecipitation with 14-3-3D (Figure 1C) and three spots (with higher molecular weights and lower isoelectric points than the unmodified protein) in a 2D western blot (Supplementary Figure S12) strengthens the idea that PTMs may be relevant for SCI1 function and/or degradation.

Using a mutagenesis strategy, through which serines of the predicted 14-3-3 binding sites of SCI1 were mutated into alanines to prevent phosphorylation, we have shown that these serines have a role in the SCI1 cell-cycle-regulated degradation. Interestingly, five out of the seven mutated serines are conserved in the Arabidopsis SCI1 sequence (Supplementary Figure S2 B). Phosphorylation-mediated signaling and the role of 14-3-3 proteins in the nucleocytoplasmic shuttling have already been demonstrated in some processes in plants, such as in the gibberellin signaling pathway (Igarashi et al., 2001) and on the shade response of PIF7 (PHYTOCHROME-INTERACTING FACTOR 7; Huang et al., 2018). 14-3-3 proteins have also been demonstrated to promote client protein degradation. For example, 14-3-3 downregulates the stability of the ETO1/EOL E3 ligase components of the ethylene signaling pathway in Arabidopsis (Yoon and Kieber, 2013). 14-3-3 proteins are also responsible for exporting phosphorylated BRZ1 and BES1 from the nucleus to the cytoplasm for proteasomal degradation (de Boer et al., 2012). Additionally, 14-3-3 proteins shuttle from the cytosol to the nucleus, where they interact with and destabilize the key cold-responsive C-repeat-binding factor (CBF) proteins (Liu et al., 2017), a mechanism similar to what we are proposing for SCI1 and 14-3-3D.

We have shown that 14-3-3D interacts with SKP1, a core component of the SCF family of E3 ubiquitin ligases (see Supplementary Figure S10). Remarkably, At14-3-3-Phi (GRF4) and At14-3-3-Upsilon (GRF5) proteins, that belong to the same Non-Epsilon group of 14-3-3D (Supplementary Figure S3), can rescue the lethality of budding yeast cells that lack the Bmh1 and Bmh2 14-3-3 proteins (van Heusden et al., 1996). Of note, Bmh1 and Bmh2 physically interact with Skp1 that, together with the SCF ubiquitin ligase complex, promotes ubiquitination and degradation of the cell-cycle inhibitor of yeast cell proliferation called Sic1 (Barberis, 2012), *in vitro* and *in vivo* (Feldman et al., 1997; Verma et al., 1997).

Evidence from budding yeast indicates that the Bmh1 and Bmh2 14-3-3 proteins are involved in regulating the spindle checkpoint (Grandin and Charbonneau, 2008; Bokros et al., 2016; Gryaznova et al., 2016). Intriguingly, the cell-cycle inhibitor Sic1 inhibits spindle pole separation (Chee and Haase, 2010) through binding and inhibiting the mitotic cyclin/Cdk1 kinases that regulate a timely cell division in budding yeast (Barberis et al., 2012; Schreiber et al., 2012). We can speculate that SCI1 may be involved in similar events by regulating mitosis in floral meristematic cells. In this hypothesis, SCI1 could interact with a protein(s) necessary for spindle-related events, inhibiting its function. Phosphorylation of SCI1 serines would release this protein, allowing its association with chromosomes and cell division to progress. However, the unphosphorylated SCI1 would remain bound to this protein and, therefore, be driven to its target site of action during cell division, explaining SCI1mut3 association with chromosomes at metaphase (Figure 6 and Supplementary Figures S8, S9).

## Conclusion and Final Remarks

The nucleoli have demonstrated roles in regulating mitosis, cell-cycle progression, and cell proliferation (Boisvert et al., 2007; Shaw and Brown, 2012). The physical containment of SCI1 in the nucleoli during interphase (Figure 3A) suggests that it may sequester and regulate the availability of cell-cycle effectors, which are necessary for cell division to progress. A similar role has been shown for Sic1, an inhibitor of the Cdk1/Clb cyclin that controls cell-cycle progression in budding yeast (Barberis, 2012; Barberis et al., 2012).

Two molecular mechanisms known to coordinate cell proliferation are phosphorylation and ubiquitin/proteasome-mediated protein degradation. Regulatory modules have evolved to integrate these two control systems at crucial decision points in the cell division cycle to manage plant development (Holt, 2012). Based on our results, we conclude that SCI1 stability is cell-cycle regulated. Its proteasomal degradation before metaphase depends on the serine residues at the predicted 14-3-3 binding sites. Therefore, we propose a model (Figure 7) in which cell division signals promote 14-3-3D translocation from the cytoplasm to the nucleus through a yet unknown mechanism. In the nucleus, 14-3-3D interacts with SCI1 and mediates its proteasomal degradation at early mitosis. SCI1, as an inhibitor of cell proliferation, would be degraded for cell division to progress appropriately. Further experiments will be required to test our proposed model.

Stigma/style cell-cycle inhibitor 1 is a regulator of cell division specifically expressed in the proliferative cells of the floral meristem (Cruz et al., 2021). So why would floral meristems have additional regulators of cell division? The development of plant organs is largely post-embryonic. However, it has already been shown that plants minimize the number of divisions of the meristematic cells that give rise to flowers and seeds to avoid passing on new mutations to their offspring (Burian et al., 2016; Watson et al., 2016). Thus, it is reasonable to assume that *SCI1* is expressed in floral meristem to allow the precise regulation (fine-tuning) of proliferation rates of the flower meristematic cells. This additional control mechanism would ensure DNA integrity passed on to the descendants, including an extra level of checking to those already existing in the other plant cells.

## Data Availability Statement

The datasets presented in this study can be found in online repositories. The names of the repository/repositories and accession number(s) can be found in the article/Supplementary Material.

## Author Contributions

ES, LB, JS, HS, FP, VP, PF, GL, and AQ conducted the research and participated in the drafting of the manuscript. Data were analyzed with the assistance of HP, L-ED-B, MM, and AA, which also revised the manuscript critically providing important intellectual contributions. NE, MB, and MG conceived the research, designed the experiments, analyzed the data, and drafted the manuscript. All authors contributed to the article and approved the submitted version.

## Funding

This work was supported by grants 2012/50562-2, 2016/20486-3, and 2019/24774-1, São Paulo Research Foundation (FAPESP)—Brazil to MG and by the Systems Biology Grant of the University of Surrey to MB. The authors are also grateful to the Brazilian agencies that financed their fellowships: CNPq to LB (130159/2012-3), JS (435447/2016-5), and VP (141909/2016-1); HS—FAPESP (2011/51844-9), and CAPES to ES, PF, and GL. Therefore, this study was financed in part by the Coordenação de Aperfeiçoamento de Pessoal de Nível Superior—Brazil (CAPES)—Finance Code 001. MG is indebted to CNPq—Brazil for her Research fellowship. MM is the PET-Ministry of Education-Brazil fellowship.

## Conflict of Interest

The authors declare that the research was conducted in the absence of any commercial or financial relationships that could be construed as a potential conflict of interest.

## Publisher’s Note

All claims expressed in this article are solely those of the authors and do not necessarily represent those of their affiliated organizations, or those of the publisher, the editors and the reviewers. Any product that may be evaluated in this article, or claim that may be made by its manufacturer, is not guaranteed or endorsed by the publisher.

## Footnotes

^1^ http://www.ncbi.nlm.nih.gov/BLAST/
