# Supplementary material for: Stigma/Style Cell-Cycle Inhibitor 1, a Regulator of Cell Proliferation, Interacts With a Specific 14-3-3 Protein and Is Degraded During Cell Division
Source: Front Plant Sci. 2022 Apr 4;13:857745. doi: 10.3389/fpls.2022.857745 (PMC9013909; doi:10.3389/fpls.2022.857745)
Supplement: Supplementary file 1 [file Data_Sheet_1.docx]

Table S1 Accession numbers of proteins used in the phylogenetic analysis

| **Species** | **Name** | **Greek name** | **Accession** |
| --- | --- | --- | --- |
| *A. thaliana* | GRF1 | Chi | L09112 |
| *A. thaliana* | GRF2 | Omega | M96855 |
| *A. thaliana* | GRF3 | Psi | L09110 |
| *A. thaliana* | GRF4 | Phi | L09111 |
| *A. thaliana* | GRF5 | Upsilon | L09109 |
| *A. thaliana* | GRF6 | Lambda | U68545 |
| *A. thaliana* | GRF7 | Nu | U60445 |
| *A. thaliana* | GRF8 | Kappa | U36447 |
| *A. thaliana* | GRF9 | Mu | U60444 |
| *A. thaliana* | GRF10 | Epsilon | U36446 |
| *A. thaliana* | GRF11 | Omicron | AF323920 |
| *A. thaliana* | GRF12 | Iota | AF335544 |
| *A. thaliana* | GRF13 | Pi | NP_565174 |
| *N. tabacum* | 14-3-3 A1 | - | AB119466 |
| *N. tabacum* | 14-3-3 B1 | - | AB119467 |
| *N. tabacum* | 14-3-3 B2 | - | AB119468 |
| *N. tabacum* | 14-3-3 C1 | - | AB119469 |
| *N. tabacum* | 14-3-3 C2 | - | AB119470 |
| *N. tabacum* | 14-3-3 D1 | - | AB119471 |
| *N. tabacum* | 14-3-3 D2 | - | AB119472 |
| *N. tabacum* | 14-3-3 E1 | - | AB119474 |
| *N. tabacum* | 14-3-3 E2 | - | AB119475 |
| *N. tabacum* | 14-3-3 F1 | - | AB119476 |
| *N. tabacum* | 14-3-3 F2 | - | AB120322 |
| *N. tabacum* | 14-3-3 G1 | - | AB119477 |
| *N. tabacum* | 14-3-3 H1 | - | AB119478 |
| *N. tabacum* | 14-3-3 H2 | - | AB119479 |
| *N. tabacum* | 14-3-3 I1 | - | AB119480 |
| *N. tabacum* | 14-3-3 I2 | - | AB119481 |
| *N. tabacum* | (TS) 14-3-3 | - | AB120323 |

Supplemental Figure S1


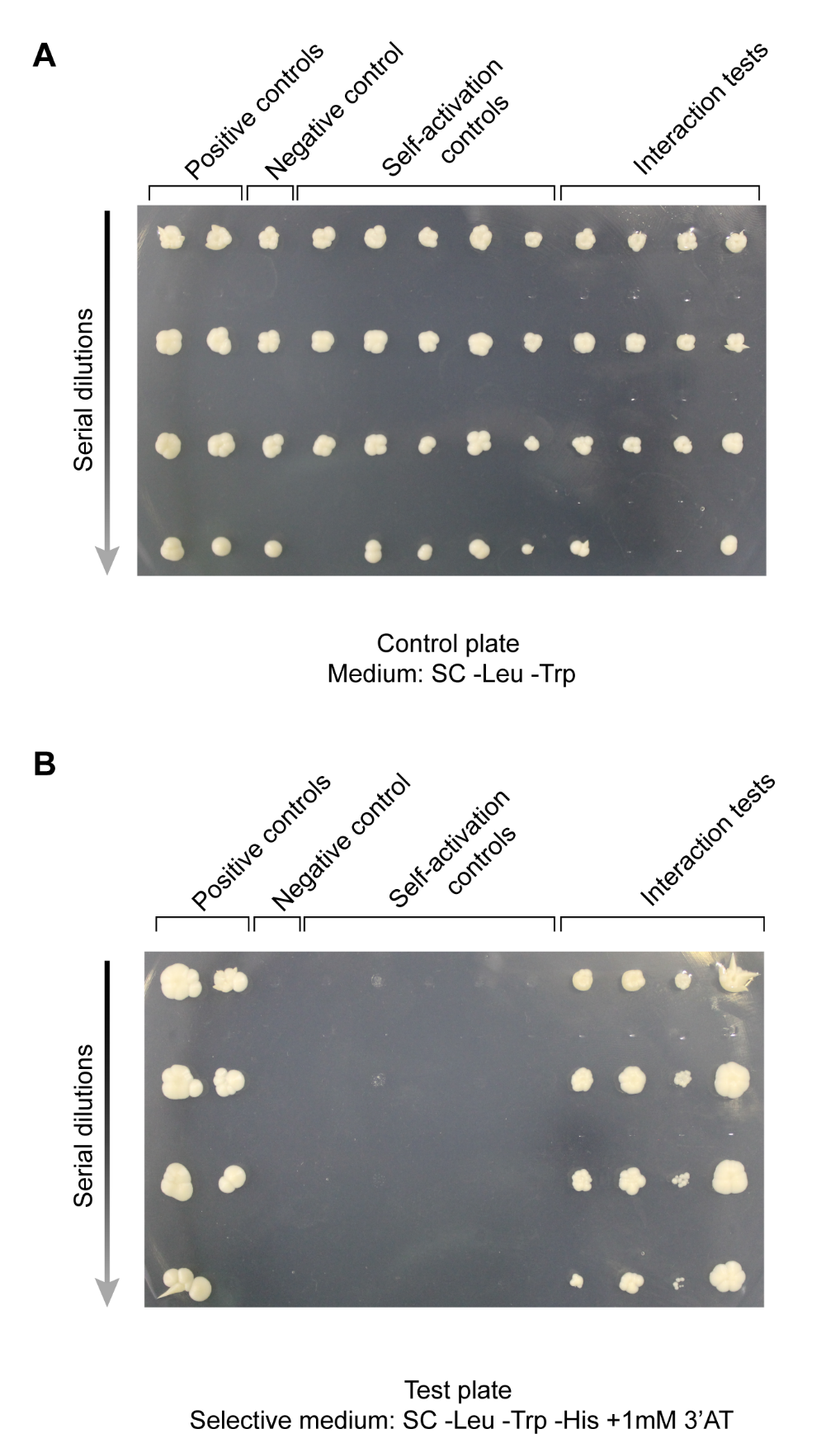


Y2H binary assays were performed in 96 well format plates.

The yeast colonies containing the relevant AD and BD constructions (for each control and interaction test) were cultured in a liquid medium (SC-Leu-Trp) overnight. Cultures were diluted to OD_600_ 0.2 with SC-Leu-Trp medium, and 250µl aliquots were placed in the first row of the 96-well plate. Subsequently, 5-fold serial dilutions were placed in the corresponding wells (the same column), of the third, fifth and seventh rows of the plate. A microplate replicator with 96 pins (Boekel) was used to make replicas on 150 mm Petri dishes containing SC solid medium.

**(A)** Control plate containing SC medium without leucine (-Leu) and tryptophan (-Trp).

**(B)** Y2H binary assays on selective medium (SC without leucine [-Leu], tryptophan [-Trp], and histidine [-His]). In the images shown, four different interactions are being tested (last four columns), and their corresponding self-activation controls were placed on the previous columns of the same plate. On the selective medium, in this case, 1mM 3’AT (3-Amino-1,2,4-Triazole) was also added. Each interaction was tested at least three times (biological replicas). After growth, the plates were photographed and images were used to prepare the final figures shown in the article.

Supplemental Figure S2


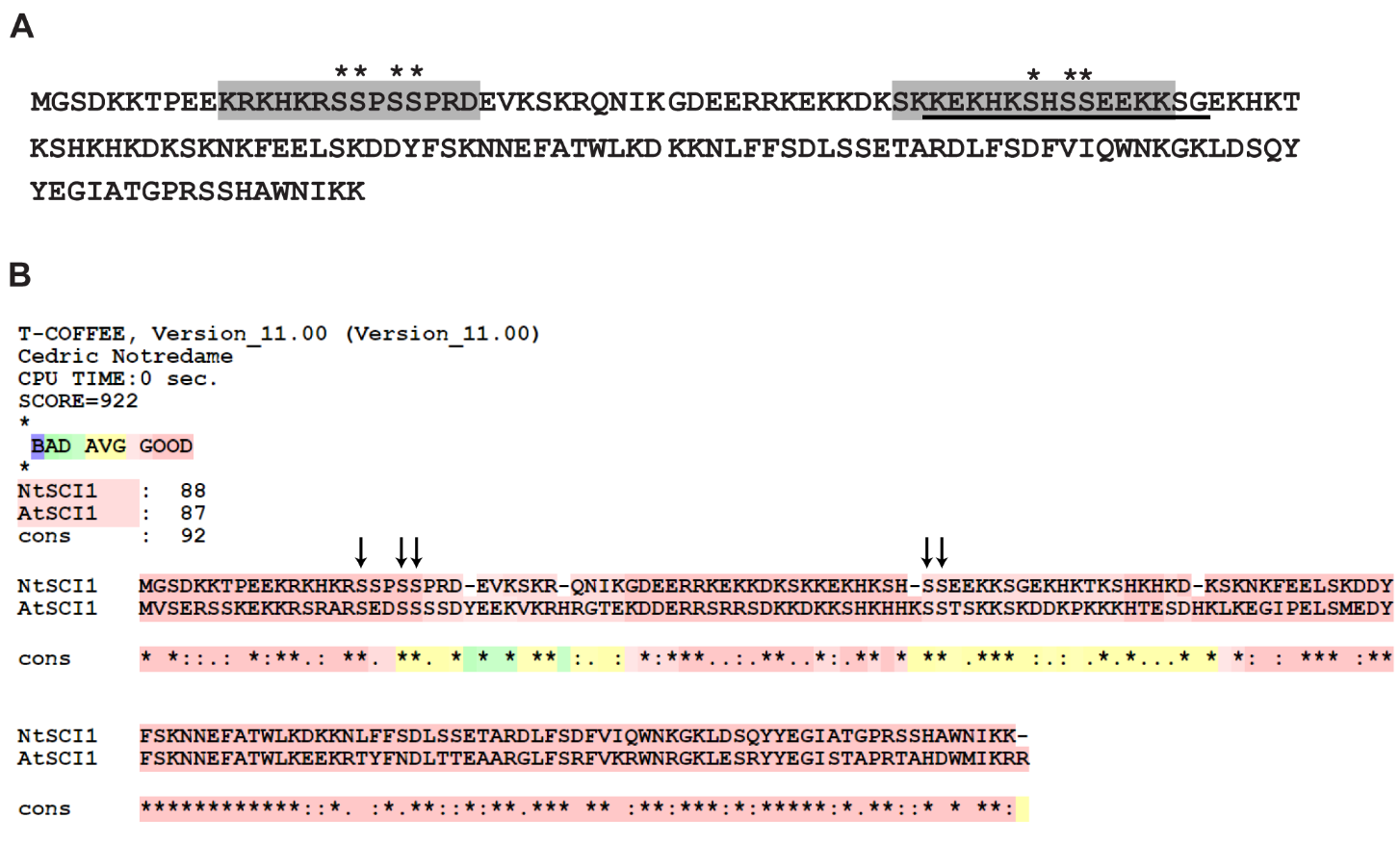


**(A)** *In silico* analysis of SCI1 by MotifScan software (Pagni et al., 2004).

14-3-3 mode I interaction motifs (n=3) were found on the SCI1 amino acid sequence. Two motifs (1 and 2) are highlighted in gray, and a third mode I motif, which overlaps with motif 2, is underlined. Asterisks indicate the serines (n=7) which were mutated into alanines. SCI1mut1 was mutated only on the first 4 serines, while SCI1mut2 had mutations only on the 3 serines of the second and third overlapping motifs. **SCI1mut3** had all the 7 serines mutated.

**(B)** Alignment of *N. tabacum* and *A. thaliana* SCI1 amino acid sequences produced by T-COFFEE (http://tcoffee.crg.cat/tcs, Chang et al., 2015). Arrows indicate the conservation of five serine residues present at the predicted 14-3-3 putative binding sites. These five serines are among the seven mutated residues in the SCI1mut3 sequence.

References:

Chang JM, Di Tommaso P, Lefort V, Gascuel O, Notredame C. (2015). TCS: a web server for multiple sequence alignment evaluation and phylogenetic reconstruction. Nucleic Acids Res. 43(W1):W3‐W6.

Pagni M, Ioannidis V, Cerutti L, Zahn-Zabal M, Jongeneel CV, Falquet L. (2004) MyHits: a new interactive resource for protein annotation and domain identification. Nucleic Acids Res. 2004 Jul 1;32(Web Server issue):W332-5.

Supplemental Figure S3


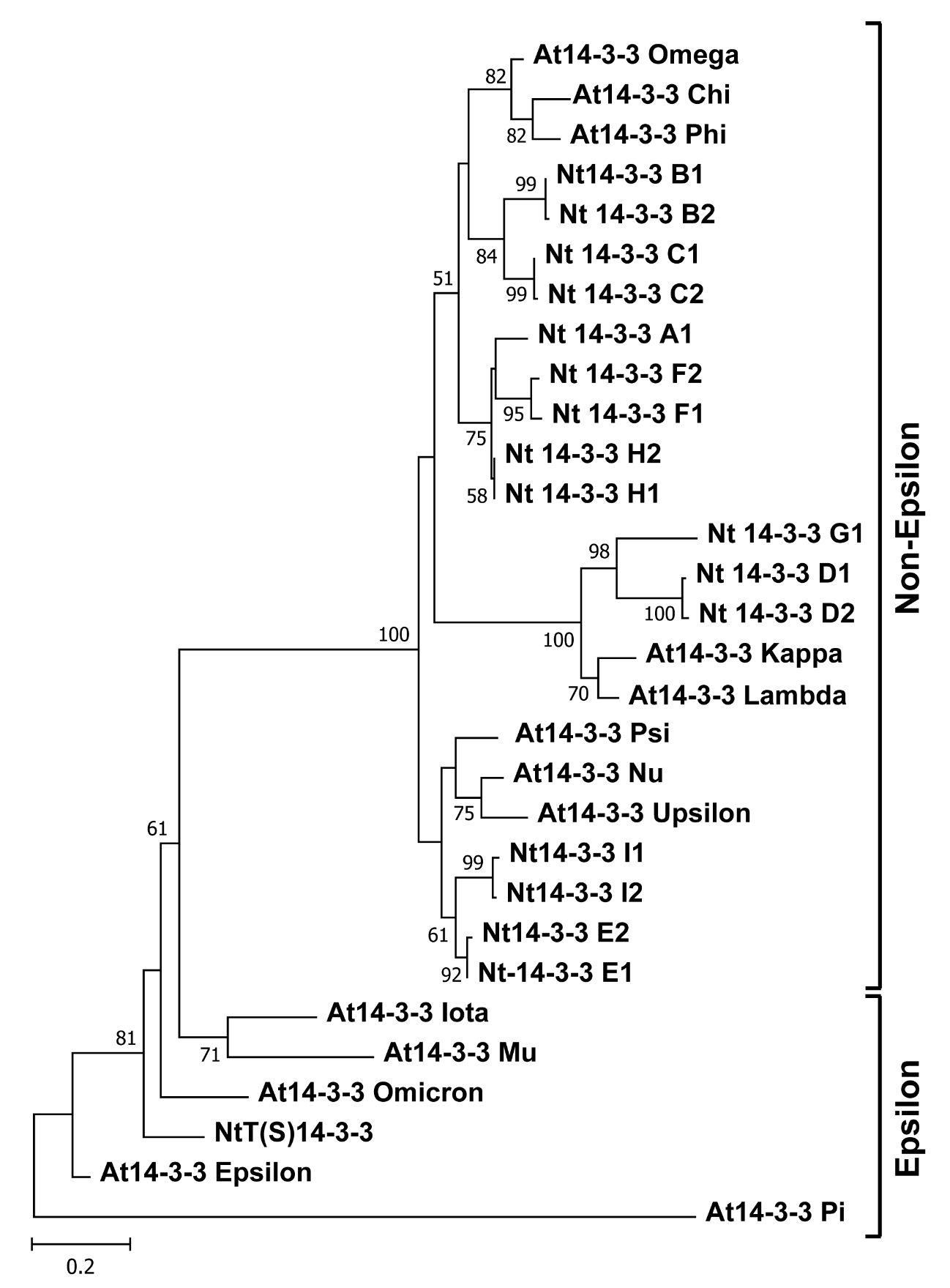


Maximum-likelihood phylogenetic tree of *A. thaliana* (At) and *N. tabacum* (Nt) 14-3-3 proteins. Bootstrap values below 50 are not shown. Scale bar indicates the expected number of substitutions per site.

Experimental Procedures

*N. tabacum* and *A. thaliana* 14-3-3 amino acid sequences (accession numbers available in Table S1) were aligned using MAFFT v7.271, with default parameters (Katoh, 2013). A maximum-likelihood phylogenetic tree was inferred using PhyML (http://www.atgc-montpellier.fr/phyml, Guindon et al., 2010), with the Jones-Taylor-Thornton substitution model with 4 categories of substitution rate. Branch support was evaluated using 1000 bootstrap replicates.

References:

Guindon S., Dufayard J.F., Lefort V., Anisimova M., Hordijk W., Gascuel O. (2010) "New Algorithms and Methods to Estimate Maximum-Likelihood Phylogenies: Assessing the Performance of PhyML 3.0. Systematic Biology, 59(3):307-21, 2010.

Katoh, Standley (2013) MAFFT multiple sequence alignment software version 7: improvements in performance and usability. Molecular Biology and Evolution 30:772-780.

Supplemental Figure S4


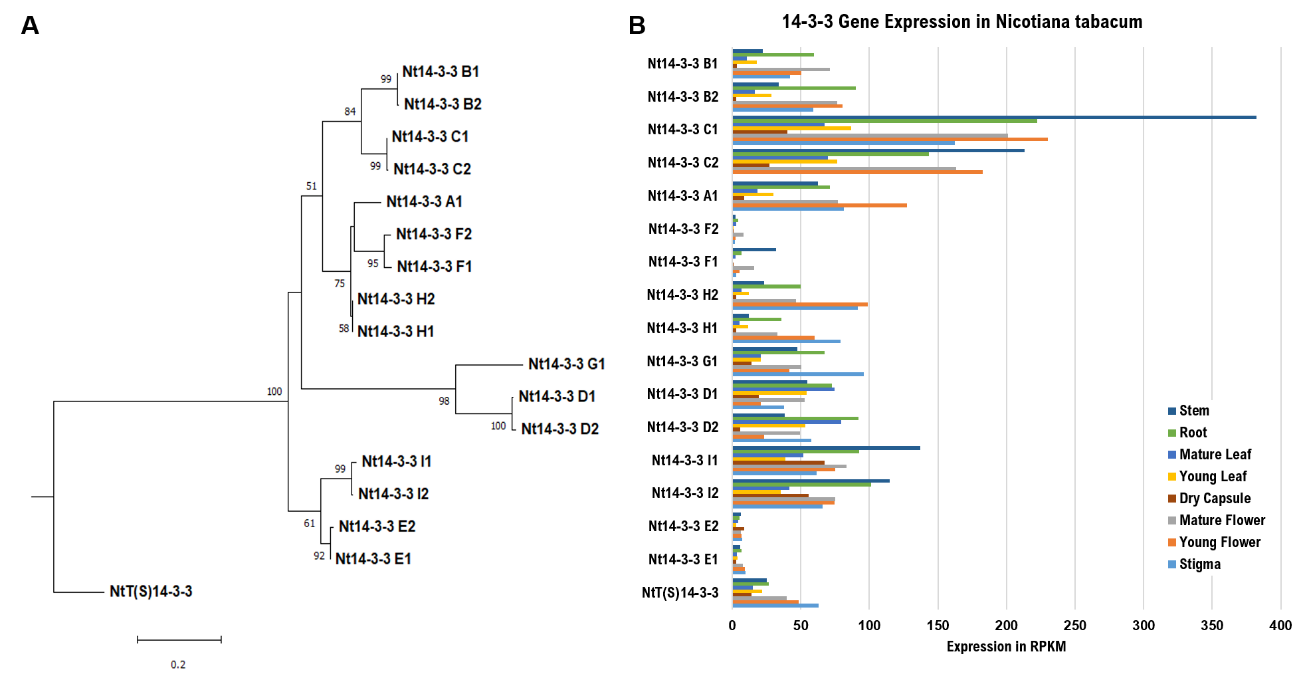


**(A)** Pruned phylogenetic tree displaying only *N. tabacum* 14-3-3 sequences (pruned version of the tree in Figure S1). Pruning of Arabidopsis sequences was achieved with the drop.tips function of the R package ape v5.3 (Paradis & Schliep, 2019).

**(B)** Expressions of *N. tabacum* 14-3-3 genes were assessed in different organs by RNA-seq, as previously described (Brito et al., 2018). Briefly, publicly available sequencing data from roots, stems, young leaves, mature leaves, young flowers, mature flowers, and dry capsules of *N. tabacum* TN90 plants, and our own stigma/style RNA-seq data were used. Reads from every sample were aligned to a reference genome of *N. tabacum* (TN90) in the CLC Genomics Workbench (Qiagen) software. The aligned reads were used to estimate gene expression in the different organs with the average of the three biological replicates for each.

References

Brito, M. S., DePaoli, H. C., Cossalter, V., Avanci, N. C., Ferreira, P. B., Azevedo, M. S., Strini, E. J., Quiapim, A. C., Goldman, G. H., Peres, L. E. P., & Goldman, M. H. S. (2018). A novel cysteine-rich peptide regulates cell expansion in the tobacco pistil and influences its final size. Plant Science, 277, 55–67.

Paradis E. & Schliep K. (2019). Ape 5.0: an environment for modern phylogenetics and evolutionary analyses in R. Bioinformatics 35: 526–528.

Supplemental Figure S5


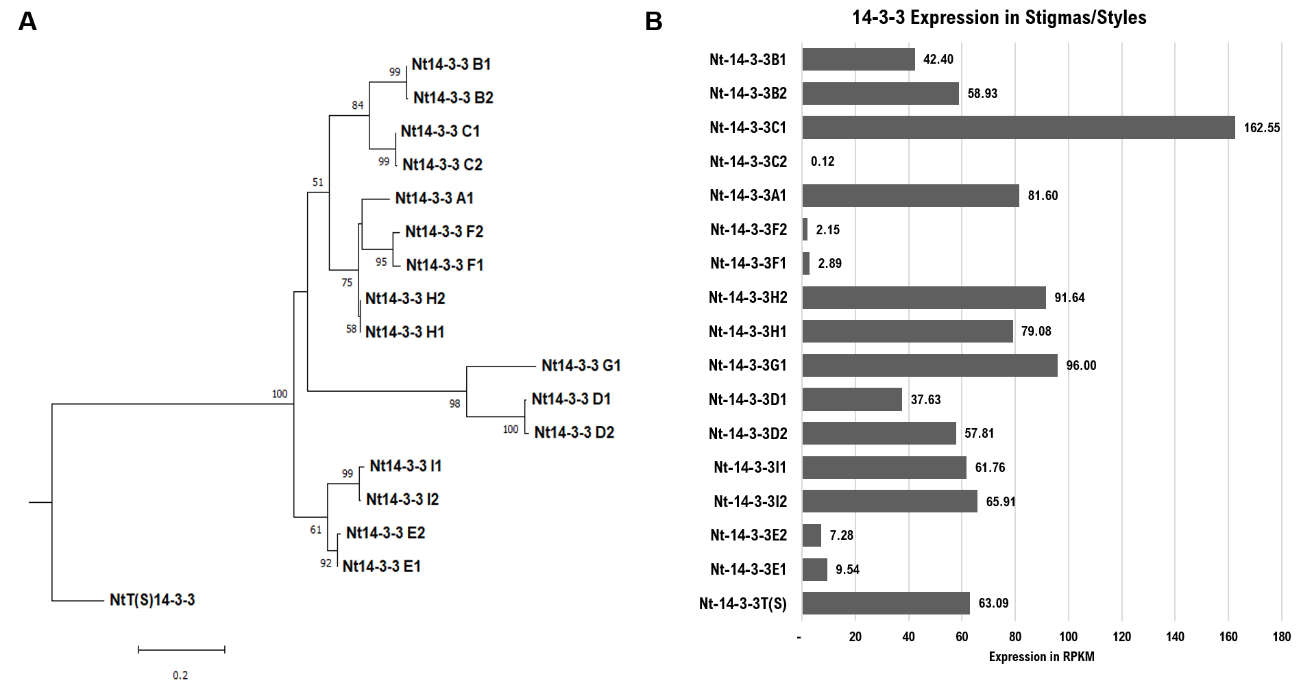


**(A)** Pruned phylogenetic tree displaying only *N. tabacum* 14-3-3 sequences (pruned version of the tree in Figure S1). Pruning of Arabidopsis sequences was achieved with the drop.tips function of the R package ape v5.3 (Paradis & Schliep, 2019).

**(B)** Expression of *N. tabacum* 14-3-3 genes were assessed in stigmas/styles by RNA-seq, as previously described (Brito et al., 2018). The aligned reads were used to estimate gene expression in stigmas/styles with the average of the three biological replicates for each.

References

Brito, M. S., DePaoli, H. C., Cossalter, V., Avanci, N. C., Ferreira, P. B., Azevedo, M. S., Strini, E. J., Quiapim, A. C., Goldman, G. H., Peres, L. E. P., & Goldman, M. H. S. (2018). A novel cysteine-rich peptide regulates cell expansion in the tobacco pistil and influences its final size. Plant Science, 277, 55–67.

Paradis E. & Schliep K. (2019). Ape 5.0: an environment for modern phylogenetics and evolutionary analyses in R. Bioinformatics 35: 526–528.

Supplemental Figure S6


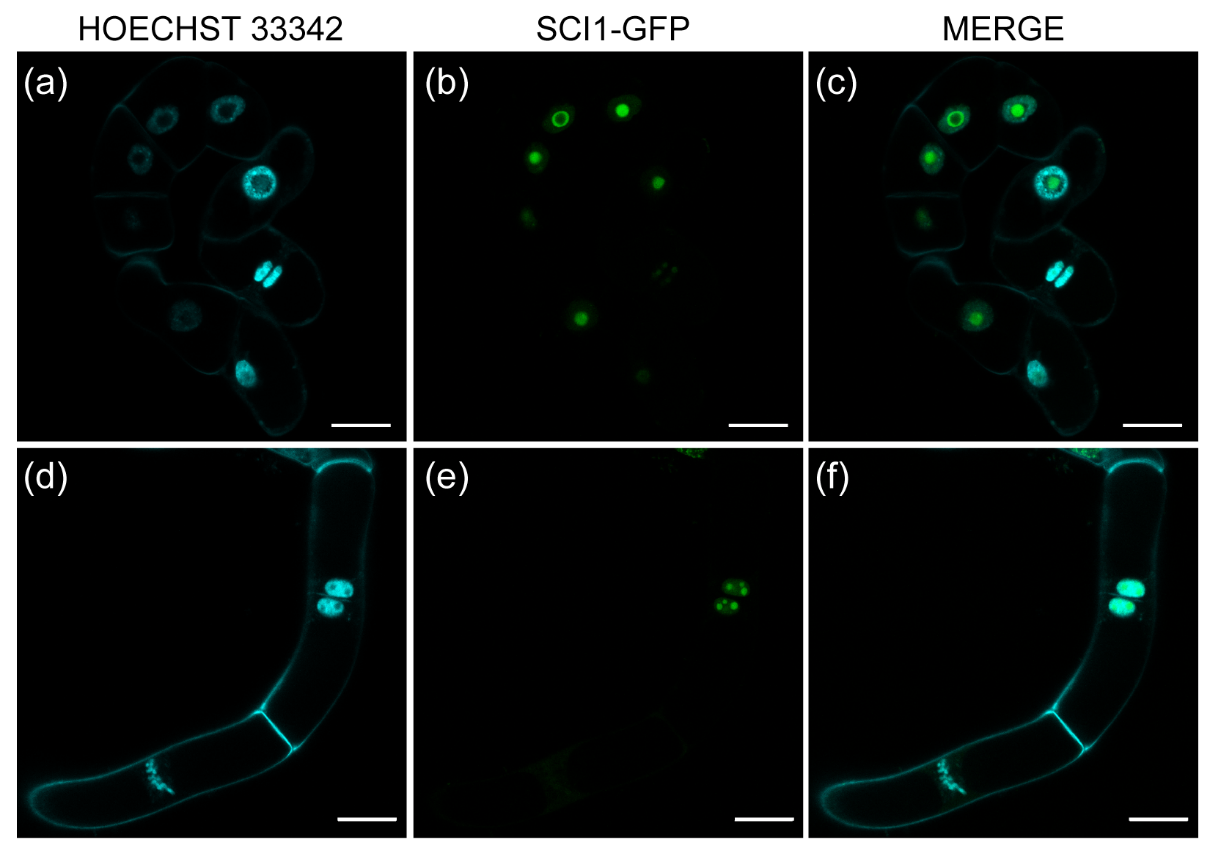


Additional images of SCI1 dynamics throughout cell-cycle progression. Confocal microscopy images showing SCI1-GFP localization in different cells of a tobacco BY-2 cell culture, stably expressing 35S_prom_::SCI1-GFP. With different cells documented in the same image, it is evident that the disappearance of SCI1-GFP in metaphase is not an artifact due to different imaging settings on the confocal microscopy. Cells were stained with Hoechst 33342 for chromatin visualization. Images were captured using Leica TCS SP5 (Leica Microsystems). Scale bars - 30 µm.

Supplemental Figure S7


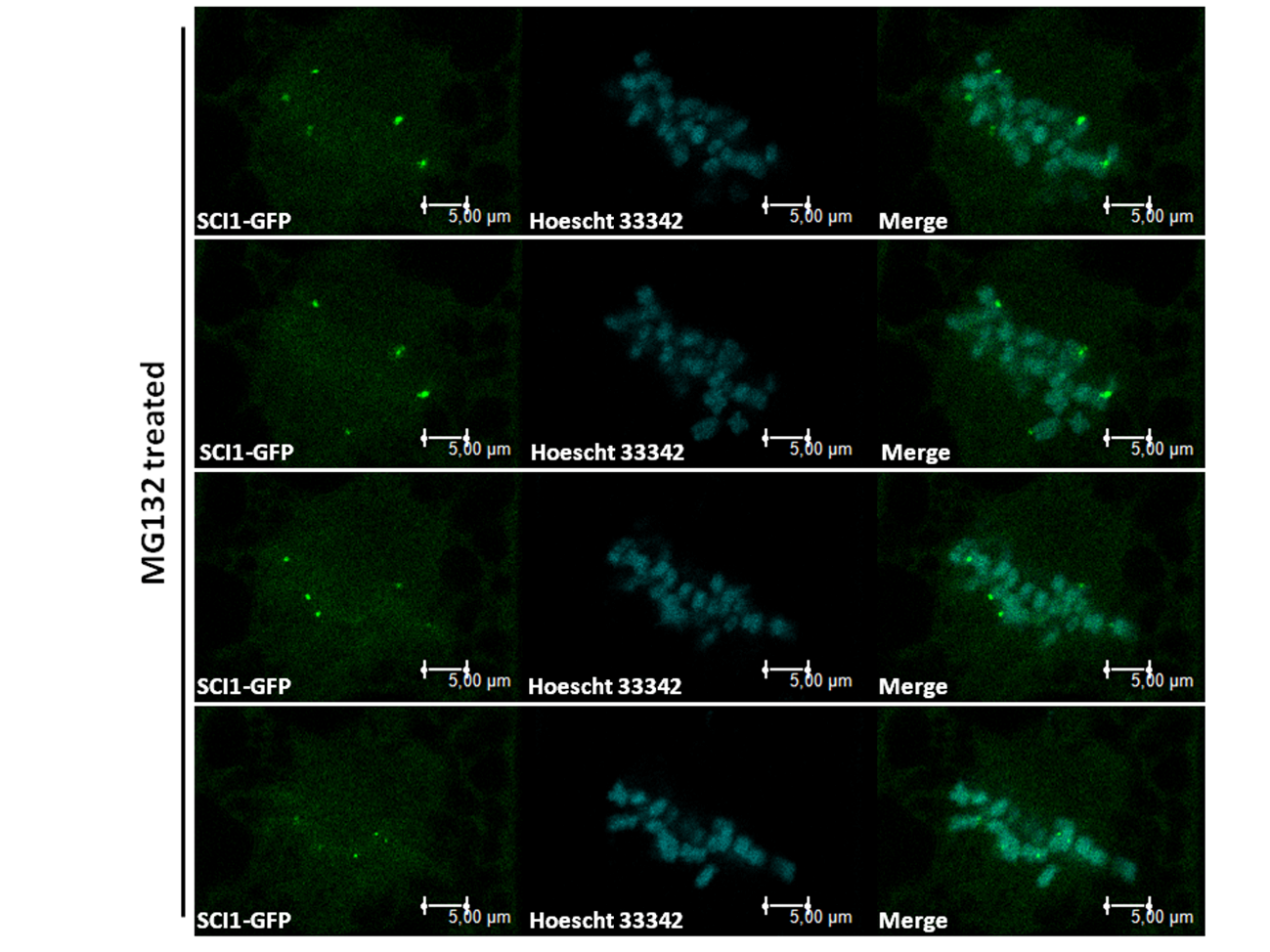


Transgenic BY-2 cells, stably expressing 35S_prom_::SCI1-GFP, treated with MG132 (50 µM in DMSO for 4 hours). Cells in metaphase show the presence of SCI1-GFP, which was stabilized by treatment with MG132, an inhibitor of 26S proteasome degradation. As shown above, SCI1-GFP is spread on the cell, but also concentrated in chromosome specific regions, which resemble Nucleolar Organizer Regions – NORs. It is possible that SCI1 localization around NORs occurs due to the fact that MG132 inhibits the degradation of several proteins, conceivably interactors of SCI1, and not only SCI1.

Supplemental Figure S8


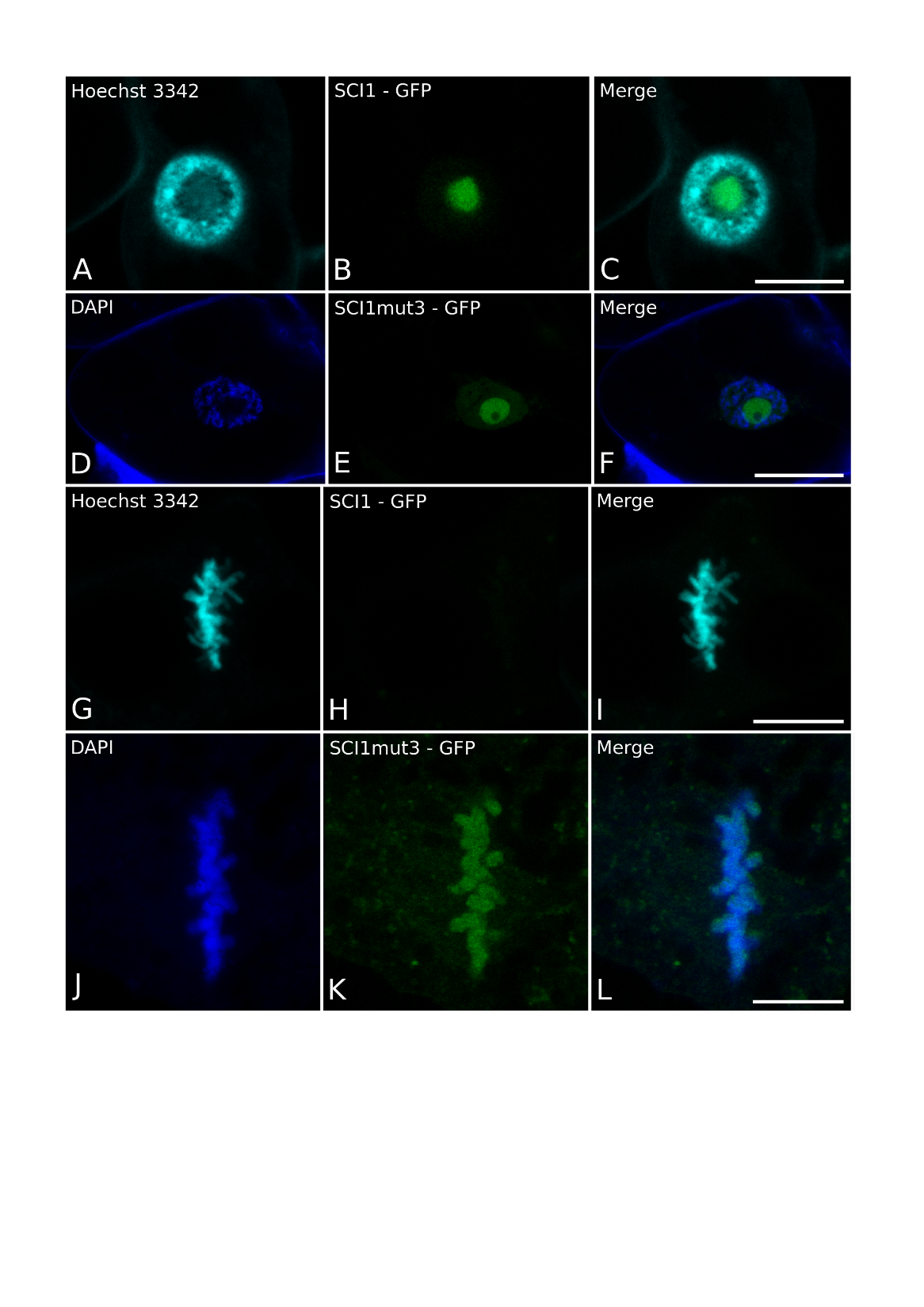


A mutated version of SCI1, in which seven serine residues were substituted by alanines (SCI1mut3), is not degraded during cell division. Confocal microscopy images showing SCI1-GFP and SCI1mut3-GFP in different cells in prophase (A-F) and metaphase (G-L). Tobacco BY-2 cells stably transformed with 35S_prom_::SCI1-GFP and 35S_prom_::SCI1mut3-GFP were stained with DAPI or Hoechst 33342, as indicated. Images were captured using Leica TCS SP5 (Leica Microsystems). Scale bars 10µm.

Supplemental Figure S9


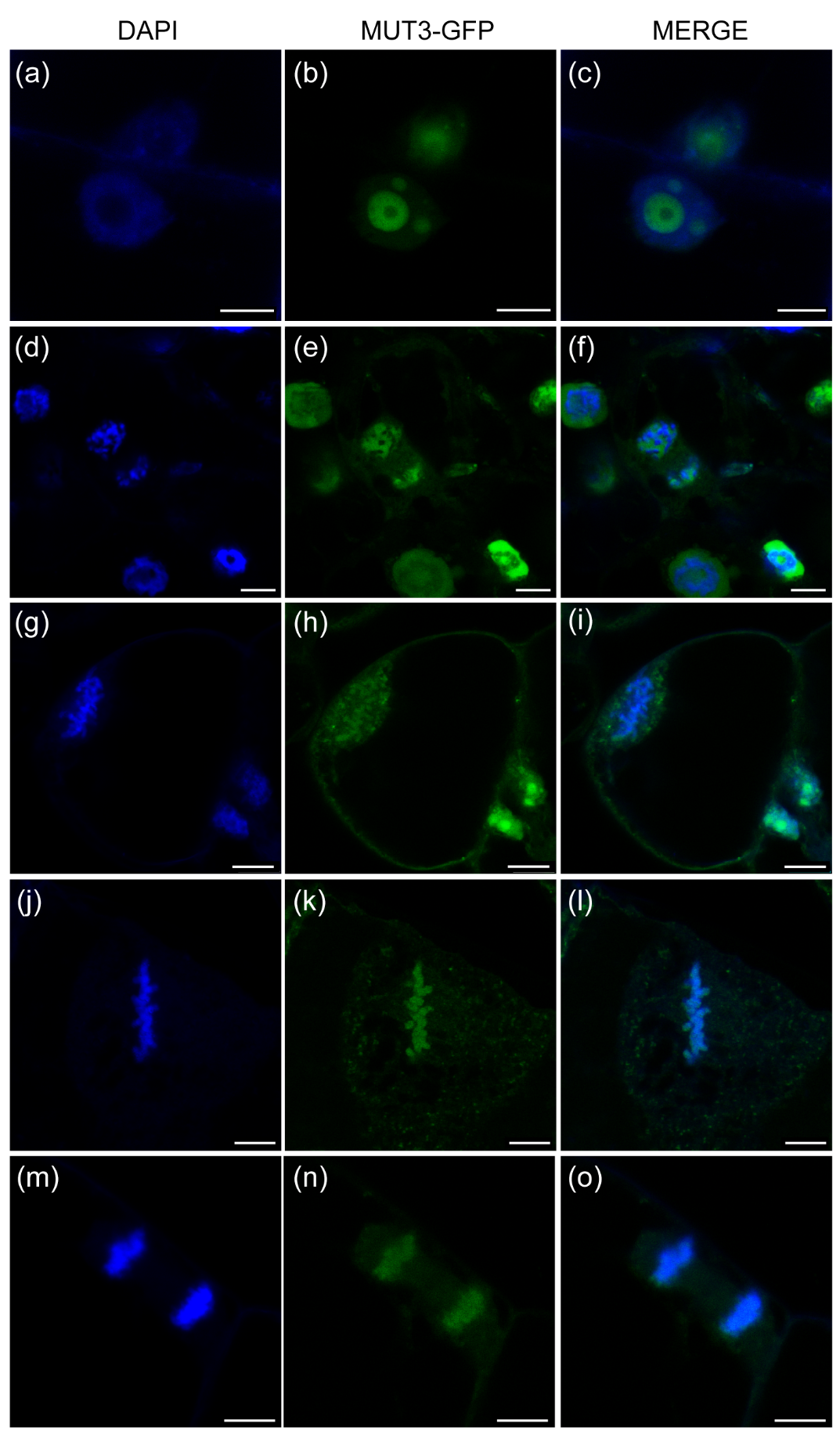


Additional images of SCI1mut3-GFP (seven serine residues mutated to alanines) expressed in stably transformed tobacco BY-2 cells (35Sprom::SCI1mut3-GFP). Here are shown cells in different stages of cell division, clearly demonstrating the SCI1mut3 is not degraded during mitosis and remains associated with chromosomes and microtubules. Cells were stained with DAPI for chromatin visualization. Images were captured using Leica TCS SP5 (Leica Microsystems). Scale bars - 10 µm.

Supplemental Figure S10


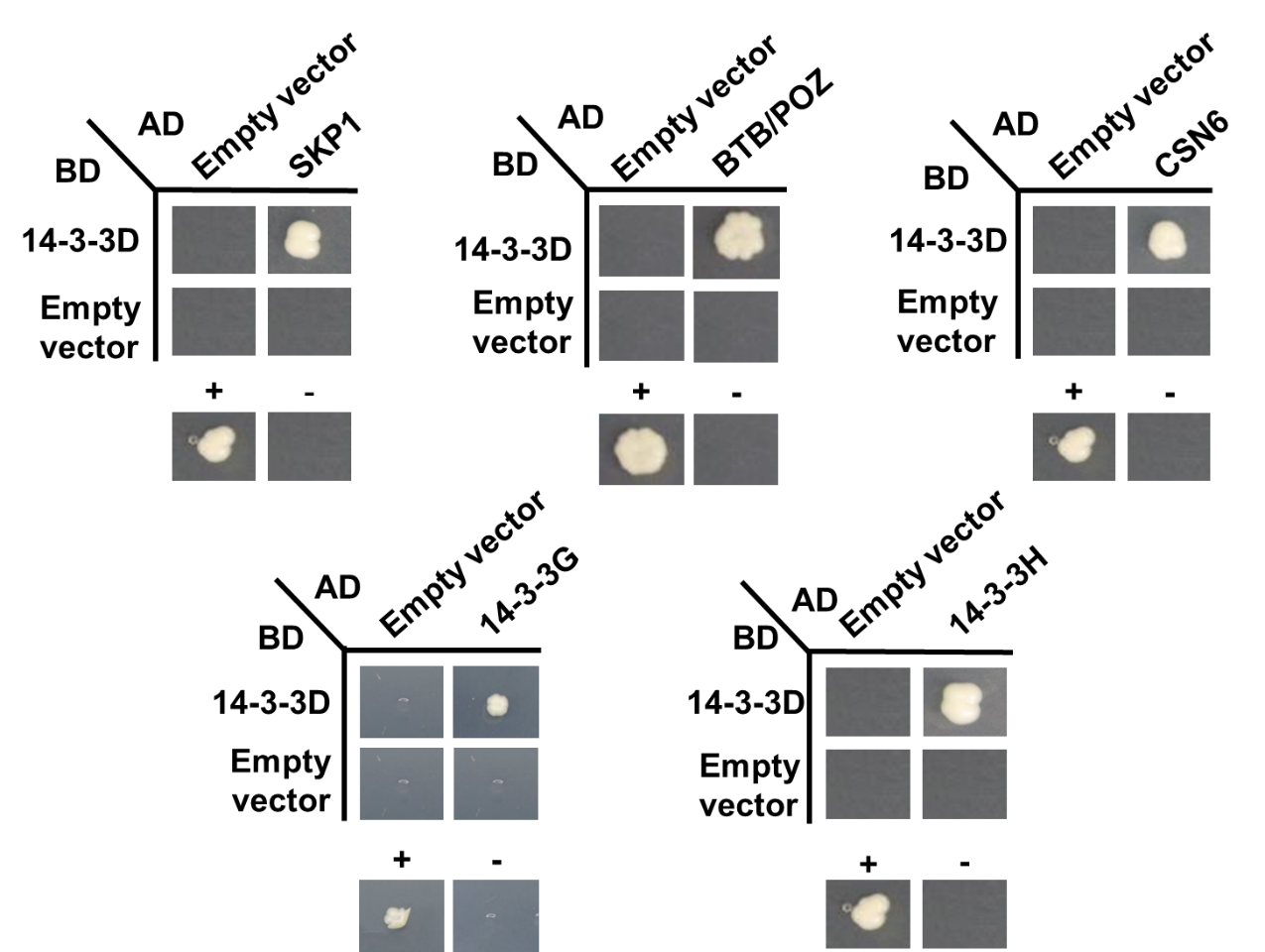


Y2H binary assays of BD-14-3-3D with some interaction partners found in the screening using 14-3-3D as bait. Yeast PJ69-4a cells with empty vectors are unable to grow on selective medium without histidine, demonstrating the absence of self-activation. The growth in culture medium lacking histidine confirms the interaction between the indicated proteins.

Note: The control images of the interactions of 14-3-3D with SKP1, CSN6 and 14-3-3H are the same, since they were performed on the same 96-well plate, as described in Materials and Methods.

Supplemental Figure S11


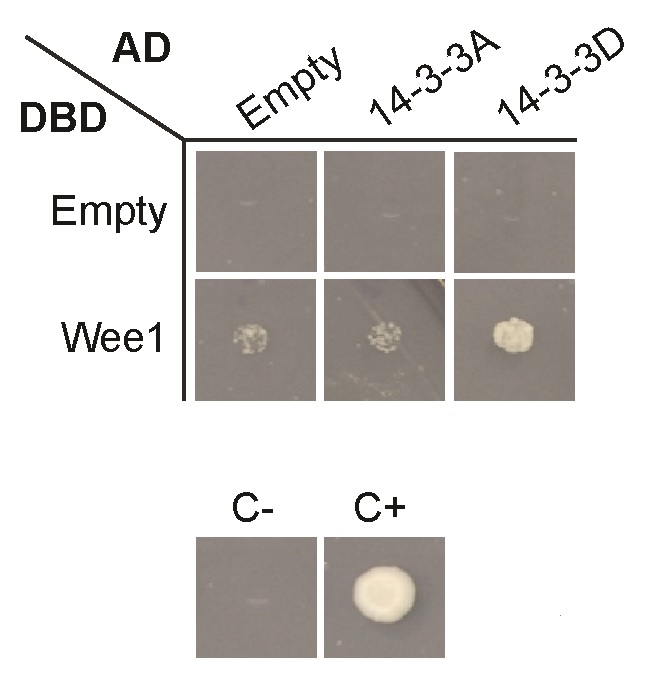


Y2H binary assay of the interaction of BD-14-3-3D with Wee1 from *N. tabacum*. Yeast PJ69-4a cells with empty vectors are unable to grow on selective medium without histidine, demonstrating the absence of self-activation. The growth in culture medium lacking histidine confirms the interaction between 14-3-3D and Wee1 proteins.

Supplemental Figure S12

**
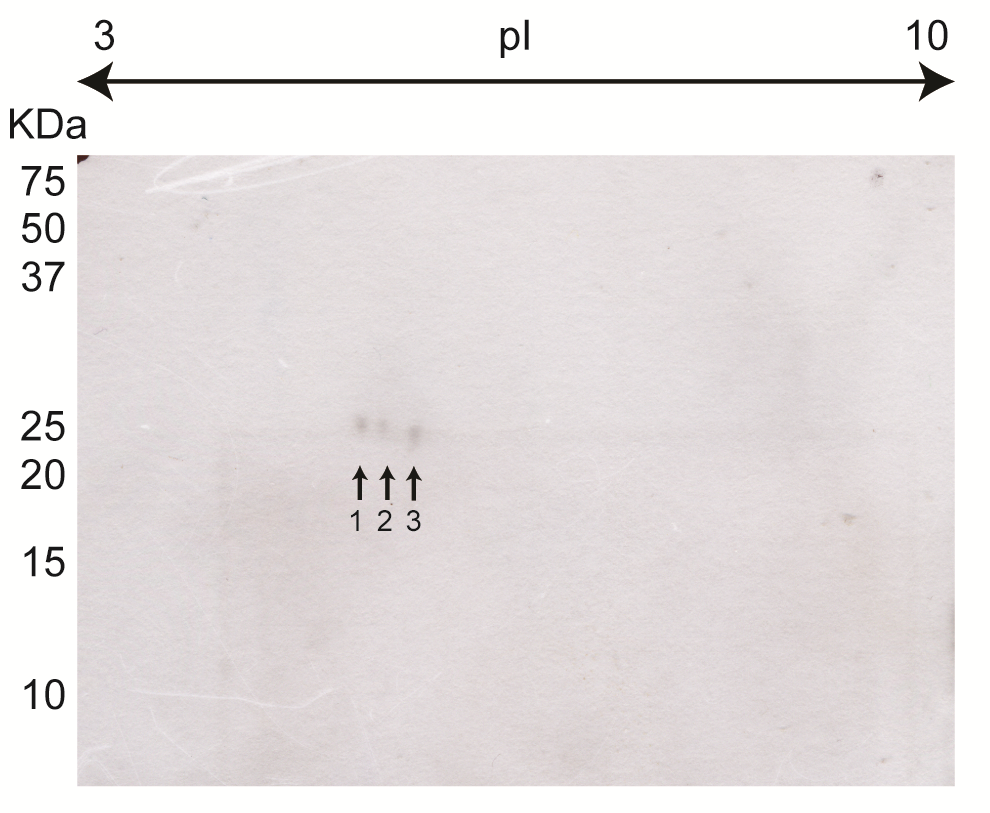
**

Western blot analysis (with anti-HIS-SCI1 polyclonal antibody) of total proteins from stigma/style of wild-type SR1 plants (100 μg) resolved by 2D gel electrophoresis.

To investigate if SCI1 is post-translationally modified in its natural cellular environment, we have extracted total proteins from stigmas/styles at stage 3 of tobacco flower development (as described by Koltunow et al., 1990), separated them by 2D gel electrophoresis and prepared a western blot. The anti-HIS-SCI1 antibody has recognized three differentially migrating spots with molecular weights between 20-25kDa and isoelectric points between 5 and 6.

Experimental Procedures

**Protein extraction from stigma/style and 2DE analysis**

Frozen sample was ground in a mortar with liquid nitrogen and incubated with extraction buffer (500 mM Tris, 50 mM EDTA, 700 mM sucrose, 100 mM KCl, pH 8.0, 2% β-mercaptoetanol and 1 mM PMSF) and vortexed (Faurobert et al. 2007). Afterward, an equal volume of Tris-buffered phenol was added and the solution was vortexed for 10 min at room temperature. The sample was centrifuged (10 min, 5500g at 4°C) and the phenolic phase was recovered. Five volumes of precipitation solution (0.1 M ammonium acetate in cold methanol) were added. The tube was inverted several times and the sample was incubated overnight at -20°C. Proteins were centrifuged (10 min, 5500g at 4°C) and the pellet was washed three times with cold acetone and vacuum dried. The pellet was resuspended (7 M urea, 2 M thiourea, 2% CHAPS, 0.3% DTT, 2% pharmalyte) and sonicated. Protein concentration was determined according to Bradford (1976). An amount of 100 µg of proteins was diluted in 150 μl rehydration buffer (7 M urea, 2% CHAPS, 2% pharmalyte, 0.002% bromophenol blue). The sample was loaded into IPGphor strip holders and overlaid with immobilized pH gradient strips (pH 3-10, 7 cm, linear gradient) that were rehydrated for 12h. IPG strips were covered with cover fluid. Isoelectric focusing (IEF) proceeded as follows: 300 V for 2 h, 1000 V to 300 kVh, 1 h, 5000 V to 4000 kVh h and 5000 V to 2000 kVh. IPG strips were incubated in equilibration buffer (75 mM Tris-HCl pH 8.8, 6 M urea, 30 % glycerol, 2% SDS, 0.002% bromophenol blue) with 1 % DTT for 20 min, then in equilibration buffer with 2.5% iodoacetamide, instead of DTT, for 30 min at room temperature. The IPG strips were placed on 14% SDS-PAGE gel and sealed in place with 0.5% agarose. Afterwards, the 2D SDS-PAGE gel was transferred to PVDF membrane (Hybond^TM^-C extra, Nitrocellulose, 45 Micron) followed by blocking with TBS-M 5% overnight. The membrane was incubated with anti-HIS-SCI1-specific antibody (produced by IgY Biotech – Brazil, against the recombinant HIS-SCI1 protein expressed in *E. coli*) in 1:400 dilution at room temperature. After washes, the membrane was incubated with Anti-Chicken IgY Peroxidase Conjugate (Sigma) in 1:3000 dilution at room temperature for 1 h. Antibodies were diluted in blocking solution and washes were done with TBS solution. Blots were visualized by using peroxidase reaction.

References:

Bradford, M.M. (1976). A rapid and sensitive method for the quantitation of microgram quantities of protein utilizing the principle of protein– dye binding. Anal. Biochem. 72, 248–254.

Faurobert, M., Pelpoir E., Chaib J. (2007). Phenol extraction of proteins for proteomic studies of recalcitrant plant tissues. Methods Mol Biol 355, 9-14.
